# Design, Synthesis, and Evaluation of Oleyl-WRH Peptides for siRNA Delivery

**DOI:** 10.3390/ph17081083

**Published:** 2024-08-18

**Authors:** Mrigank Shekhar Rai, Muhammad Imran Sajid, Jonathan Moreno, Keykavous Parang, Rakesh Kumar Tiwari

**Affiliations:** 1 Center for Targeted Drug Delivery, Department of Biomedical and Pharmaceutical Sciences, Harry and Diane Rinker Health Science Campus, Chapman University School of Pharmacy, Irvine, CA 92618, USA; mrrai@chapman.edu (M.S.R.); msajid@chapman.edu (M.I.S.); jonmoreno@chapman.edu (J.M.); 2 Faculty of Pharmacy, University of Central Punjab, Lahore 54000, Pakistan; 3 Department of Basic Medical Sciences, College of Osteopathic Medicine of the Pacific–Northwest, Western University of Health Sciences, Lebanon, OR 97355, USA

**Keywords:** acylation, arginine, breast cancer, cell-penetrating peptide, histidine, MDA-MB-231, oleic acid, RNA interference, siRNA, SK-OV-3, STAT-3, tryptophan, Western blotting

## Abstract

Delivering nucleic acid therapeutics across cell membranes is a significant challenge. Cell-penetrating peptides (CPPs) containing arginine (R), tryptophan (W), and histidine (H) show promise for siRNA delivery. To improve siRNA delivery and silence a model STAT3 gene, we hypothesized that oleyl acylation to CPPs, specifically (WRH)_n_, would enhance STAT3 silencing efficiency in breast and ovarian cancer cells. Using Fmoc/tBu solid-phase peptide chemistry, we synthesized, purified, and characterized the oleyl-conjugated (WRH)_n_ (n = 1–4) peptides. The peptide/siRNA complexes were non-cytotoxic at N/P 40 (~20 μM) against MDA-MB-231, MCF-7, SK-OV-3, and HEK-293 cells after 72 h incubation. All peptide/siRNA complexes showed serum stability at N/P ≥ 40. The synthesized conjugates, with a diameter of <100 nm, formed nano-complexes with siRNA and exhibited a stable range of zeta potential values (13–18 mV at N/P = 40). Confocal microscopy and flow cytometry analysis provided qualitative and quantitative evidence of a successful cellular internalization of siRNA. The peptides oleyl-(WRH)_3_ and oleyl-(WRH)_4_ showed ~60% and ~75% cellular uptake of siRNA, respectively, in both MDA-MB-231 and SK-OV-3 cells. Western blot analysis of oleyl-(WRH)_4_ demonstrated effective silencing of the STAT-3 gene, with ~75% silencing in MDA-MB-231 cells and ~45% in SK-OV-3 cells.

## 1. Introduction

Traditional chemotherapeutics for treating cancer suffer a major setback due to their widespread side effects and emerging resistance. Cancer poses a burgeoning health burden. There is an increasing demand for finding new and effective strategies that could bypass the side effects of and resistance to current therapeutics. One hallmark of many cancer cells is their specific genetic alterations [1].

One promising approach attracting the health care community’s attention is small interfering RNA (siRNA)-mediated gene silencing, a type of RNA-based therapeutics. The effectiveness of RNA-based gene therapy hinges on the intracellular functionality of therapeutic RNA within target cells. Categorized based on their biochemical mechanisms, these therapies require careful consideration of a clinically significant drug delivery method [2]. The Food and Drug Administration (FDA) approved the first siRNA drug 20 years after the first report on RNAi in eukaryotic cells. Recently, the FDA and the European Medicines Agency (EMA) approved siRNA therapeutics, including patisiran for hereditary transthyretin-mediated amyloidosis (hATTR), givosiran for acute hepatic porphyria, lumasiran for primary hyperoxaluria type 1, and inclisiran for hypercholesterolemia [3]. Additionally, the FDA has accepted a new drug application for vutrisiran, targeting polyneuropathy associated with hATTR amyloidosis [2,3].

siRNA has been delivered using various drug delivery approaches, including polymers [4], aptamers [5], lipid nanoparticles [6], viral carriers [7], and dendrimers [8,9]. However, these techniques still have limitations, such as cargo-carrying capacity, low delivery efficiency, the risk of mutation, high cytotoxicity, and lack of target specificity [10]. A possible alternative siRNA delivery system is afforded by the use of appropriate cell-penetrating peptides (CPPs).

CPPs containing cationic amino acids such as lysine and arginine (R) and hydrophobic residues such as tryptophan (W), alanine, or phenylalanine have been used for siRNA delivery [11,12,13]. Previous studies from our research group demonstrated that the acylation of CPPs with suitable fatty acyl chains enhanced the delivery of macromolecules such as siRNA by providing a hydrophobic region that aids in the stabilization and protection of these molecules during delivery. This improvement facilitates efficient cellular uptake and reduces degradation [13,14,15]. Furthermore, the positively charged residues, such as R, of CPPs can interact with the negatively charged backbone of siRNAs [14]. Various combinations of R and W in CPPs have shown promise in intracellular siRNA delivery [15,16]. Previous data indicated that oleyl-conjugated CGRKR peptides can deliver siRNA into tumor cells due to enhanced hydrophobicity [17,18]. A follow-up study from our laboratories demonstrated the application of histidine (H) and R in CPPs with an oleyl chain in delivering siRNA with promising cellular uptake and gene silencing efficiency in triple-negative breast cancer cells compared with standard transfecting agents, e.g., lipofectamine [18].

**Figure 1:**
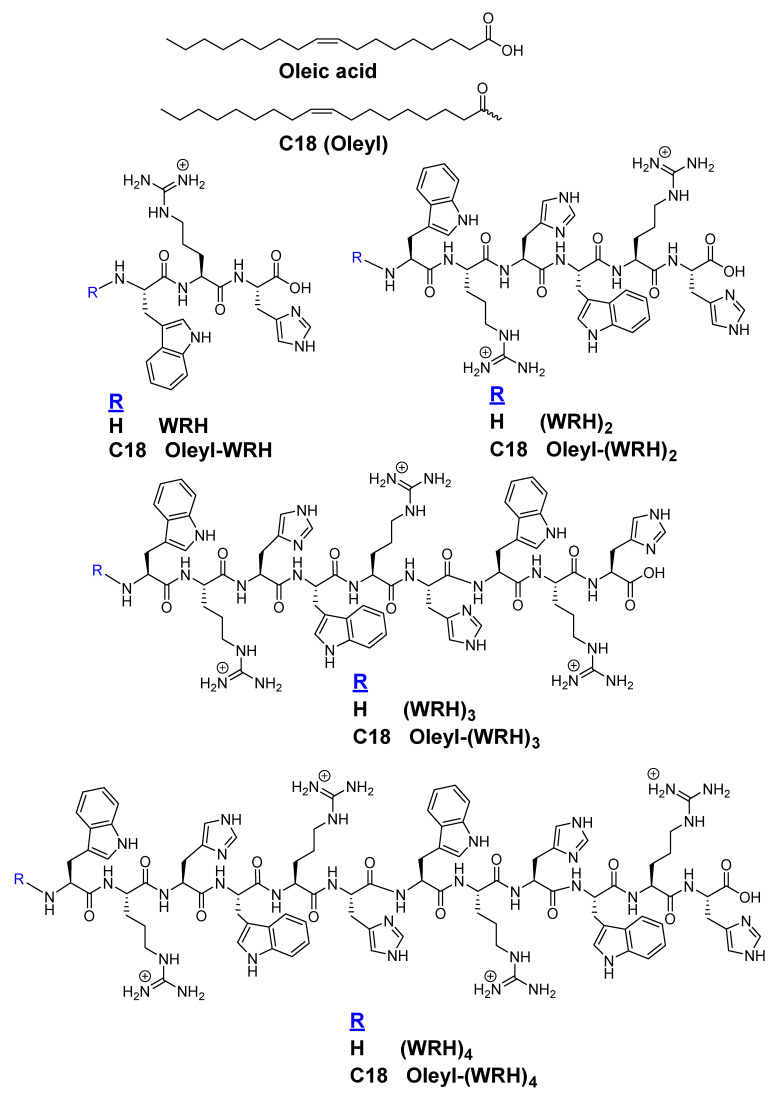
Structure of synthesized peptides. Oleyl refers to the fatty acyl chain of oleic acid, C18.

It has been observed that unsaturated fatty acids also enhance membrane permeability. The presence of R residue provides strong binding interactions to siRNA, whereas H residue helps create a proton sponge effect in endosomes, aiding in endosomal escape. H residue was selected due to its ability to utilize its imidazole ring as an endosome destabilization mechanism, facilitating the delivery of siRNA into the cytosol. The imidazole ring of histidine acts as a weak base, acquiring a cationic charge when the local pH environment drops below pH 6 [19]. The addition of W residues enhances the amphiphilic properties of peptides. Therefore, we hypothesize that fatty acylation of a (WRH)_n_ peptide with an oleyl chain (n = 1–4) (Figure 1) could enhance siRNA delivery.

The goal of this study is to design and evaluate oleyl-conjugated (WRH)_n_ peptides as effective non-toxic carriers for the intracellular delivery of siRNA. We used STAT-3 siRNA as a model to investigate the efficiency of CPP-based siRNA delivery. The use of linear WRH peptides with fatty acyl chains has not yet been explored in drug delivery. To the best of our knowledge, there has been no previous report demonstrating the use of linear peptides constituting oleic acid with W, R, and H residues for siRNA delivery in MDA-MB-231 and SK-OV-3 cells. Therefore, we designed linear (WRH)_n_ peptides (n = 1–4) with or without oleic acid chains.

In this study, we synthesized Oleyl-WRH peptides as a siRNA delivery system using solid-phase peptide synthesis and evaluated their cytotoxicity, cellular uptake of peptide/siRNA complexes, and STAT3 gene silencing efficiency. It is believed that amino acids with hydrophobic properties, like W, can generate a hydrophobic region and provide amphiphilicity to the peptide. Our studies will generate data to provide the feasibility of using appropriate oleyl-WRH peptides for siRNA delivery.

## 2. Results and Discussion

### 2.1. Chemistry

**Scheme 1:**
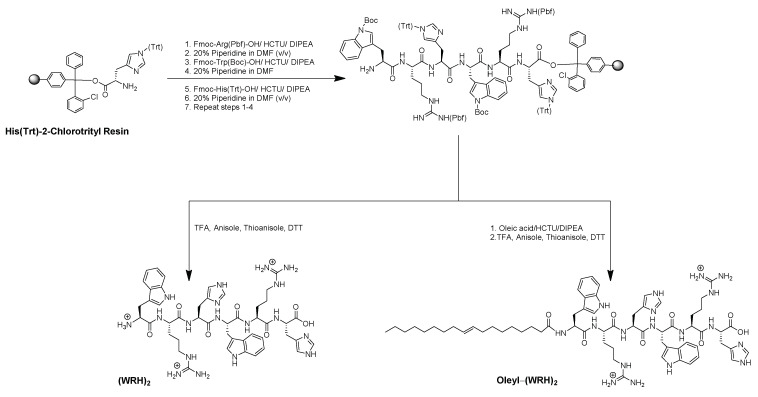
A synthetic scheme of (WRH)_2_ and Oleyl-(WRH)_2_ peptides.

The peptides were synthesized using a solid-phase peptide synthesis strategy by reacting Fmoc-protected building block amino acids on the free amino group at the His-(Trt)-Cl-2-chlorotrityl resin. Peptide synthesis was accomplished and optimized by using three equivalents of Fmoc-protected amino acids and 2-(6-chloro-1H-benzotriazole-1-yl)-1,1,3,3-tetramethylammoniumhexafluorophosphate/*N*,*N*-diisopropylethylamine (HCTU/DIPEA) in *N*,*N*-dimethylformamide (DMF) (Scheme 1 and Table 1). After the assembly of synthesized peptides, they were cleaved using a freshly prepared cleavage cocktail and were characterized using matrix-assisted laser desorption ionization mass spectroscopy (MALDI) analysis, which confirmed their molecular weights under the positive linear mode of MALDI mass spectrometry. Reversed-phase high-performance liquid chromatography (RP-HPLC) was employed for the purification of crude peptides, followed by lyophilization to obtain a dry white powder for all synthesized peptides showing ≥ 95% purity. Then, oleic acid was conjugated to the peptides using a similar procedure with a reaction with fully protected peptide-attached resin before cleavage and deprotection. However, the coupling time was increased compared with coupling amino acids on peptidyl resin. A Kaiser test was used to verify that the coupling process was fully completed and all *N*-terminal amino groups had been reacted. The hydrophobicity of the peptides varied based on the number of W residues and the presence of an alkyl chain from oleic acid acylation at the *N*-terminus. The synthesis and purification of oleyl-(WRH)_4_ was challenging owing to its increased hydrophobicity due to the presence of four W residues and one oleyl chain. This led us to fix the solubility issue of the oleyl-conjugated peptides by dissolving them using an excess mixture of solvent (acetonitrile: H_2_O, 70:30 *v*/*v*). All synthesized peptides were characterized using high-resolution MALDI mass spectrometry, confirmed to have a purity of ≥95% using analytical HPLC, and subsequently used in bioassays. (Supplementary Materials)

### 2.2. Zeta Potential and Particle Size Analysis

**Figure 2:**
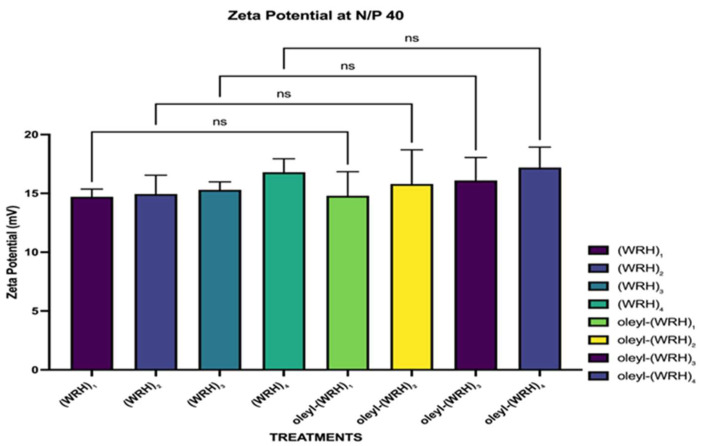
Zeta potential values of peptide: siRNA complexes at an N/P ratio of 40. Two-way ANOVA was performed using Tukey’s multiple comparison test to determine the effect of changing the N/P ratio on a zeta potential; ns = non-significant.

Zeta potential demonstrates the complexes’ net surface charge, which is crucial for their stability and interactions with biological systems. We used the Smoluchowski model to calculate the zeta potential values. To ensure precision and dependability, all measurements were performed at 40 V using disposable folded capillary cells in duplicate, with 20 repeats per measurement. The N/P ratio represents the molar ratio of protonatable nitrogen (N) atoms found in cationic components of peptide to anionic phosphate groups (P) in siRNA. The data below indicate that the peptide/siRNA complexes show an overall positive zeta potential value ranging from ~13 mV to ~18 mV at an N/P ratio of 40 (Figure 2), demonstrating relatively stable complexes (Table 2). The increasing number of R residues led to a slight enhancement in the zeta potential values.

**Figure 3:**
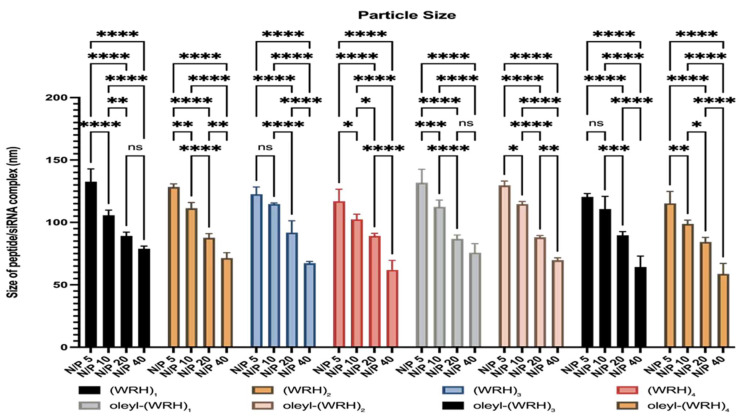
Particle size evaluation of peptides (WRH)1 to oleyl-(WRH)4 at different N/P ratios. Two-way ANOVA was performed using Tukey’s multiple comparison test to determine the effect of changing the N/P ratio on the particle size; ns = non-significant (*p*-value > 0.05), * *p*-value < 0.05, ** *p*-value < 0.01, *** *p*-value < 0.001, and **** *p*-value < 0.0001. Error bars represent the standard deviation (*n* = 3).

Particle size analyses were performed using automatic attenuator settings at the same N/P ratios and siRNA doses as dynamic light scattering (DLS) analysis. This investigation intended to examine the complexes’ size features in more detail. Lower values of the polydispersity index (PDI), a measurement of nanosuspension’s physical stability, indicate a more desired, restricted size distribution. A PDI score above 0.5 indicates a broad distribution, whereas a score between 0.1 and 0.25 indicates a somewhat tight distribution. As demonstrated in Table 3, the PDI values in this investigation of about 0.2 suggested minimum aggregation of the complexes. The particle size of all the synthesized non-oleyl-conjugated and oleyl-conjugated peptide complexes was observed to range from ~58 nm to ~79 nm at an N/P ratio of 40 (Figure 3, Table 3). The observable decline in a trend may be attributed to the rising presence of R residues and the presence of an oleic acid fatty acyl chain.

All peptides formed stable nano-complexes with siRNA with a hydrodynamic diameter < 79 nm at an N/P ratio of 40 and positive zeta potential values, which are important attributes for a successful siRNA delivery system. The ability of the peptides to form complexes within the nano-range could positively contribute to the intracellular uptake of siRNA in various cells. Figure 3 shows a particle size evaluation of peptide:siRNA at different N/P ratios, suggesting reduced size at higher N/P ratios with a higher concentration of positively charged residues, presumably with a higher interaction with negatively charged siRNA.

### 2.3. Gel Electrophoresis for Binding Affinity

**Figure 4:**
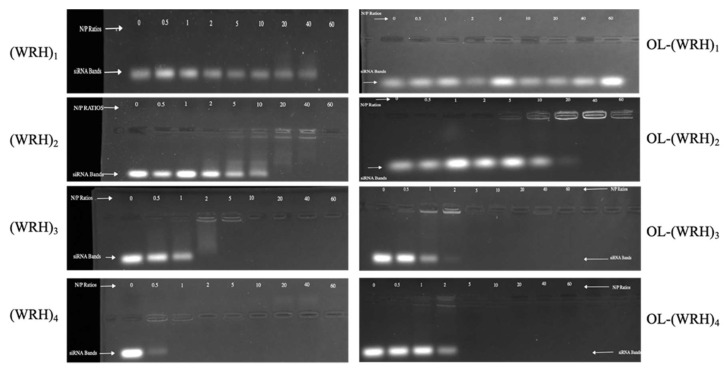
Representative agarose gel images of gel electrophoresis assay for evaluating the binding affinity of the non-oleyl-conjugated and oleyl-conjugated peptides/siRNA complexes at different N/P ratios using scrambled siRNA.

Gel electrophoresis was used to measure the binding affinity between the oleyl-conjugated peptides and scrambled siRNA. The complexes were created with final siRNA concentrations of 36 nM at various N/P ratios ranging from 0 to 60. The next step was conducting 1% agarose gel electrophoresis, followed by ethidium bromide staining. A 1% agarose gel has been proven to be optimal for elucidating the siRNA band. Using Image LabTM software 6.0.1, the bands that represented unbound siRNA were measured for intensity. This assay enabled us to evaluate the extent to which the peptides bind and safeguard siRNA during delivery. All the peptide conjugates except (WRH)_1_ and oleyl-(WRH)_1_ showed adequate retardation of siRNA at an N/P ratio ≥ 40 (Figure 4). At an N/P ratio of 0, all the siRNA was represented as a thick band, but the increase in peptide concentration in the complexes (N/P ratio) led to an increase in the binding of siRNA, meaning 100% of siRNA bound at an N/P ratio ≥ 40. The siRNA bound with oleyl-conjugated peptide was represented by the decrease in band intensity, thereby retarding the movement of siRNA in the gel, as shown in the images in Figure 4. siRNA bands start to disappear at different N/P ratios for different peptides, which means that siRNA completely binds with the peptide at that specific N/P ratio. According to Figure 4, peptides (WRH)_1_ and oleyl-(WRH)_1_ did not bind with siRNA up to an N/P ratio of 60, which is a very low binding affinity as compared with that of the other peptides in the pool. As the number of R residues increased, the peptide:siRNA binding was observed. The presence of R residues mostly correlated well with the increase in binding affinity of the non-oleyl- and oleyl-conjugated peptides towards siRNA.

### 2.4. Serum Stability of Peptide/siRNA Complexes

The peptide/siRNA complexes were exposed to a 25% (*v*/*v*) fetal bovine serum (FBS) solution in Hanks’ Balanced Salt Solution (HBSS) to evaluate the protective qualities of oleyl-conjugated peptides against enzymatic degradation. The final concentration of siRNA used in the complexes was 36 nM, while the N/P ratios used in their preparation ranged from 0 to 60. An N/P ratio of 0 means that the experimental solution contains only the fixed amount of siRNA (36 nM) with no peptide. As the N/P ratio increases, the concentration of the peptides also increases accordingly (see Section 4.3 for further details on complex formation). The heparin competition assay was used to separate the peptide/siRNA complexes and determine how much intact siRNA was present after the complexes had been incubated at 37 °C for 24 h. This assay offered a trustworthy method for evaluating the stability of the complexes and the level of resistance to enzymatic degradation provided by the oleyl-conjugated peptides. The intensity of the bands corresponding to intact siRNA was measured following gel electrophoresis using ethidium bromide staining.

**Figure 5:**
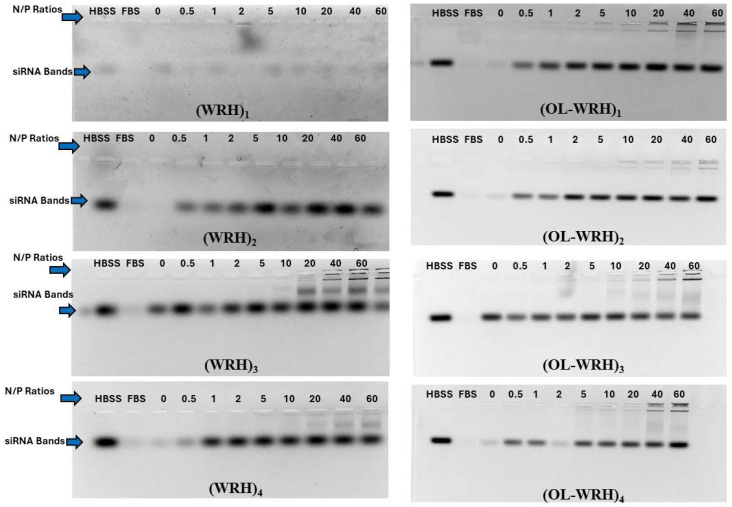
Representative agarose gel images of gel electrophoresis assay for evaluating the serum stability of the non-oleyl-conjugated and oleyl-conjugated peptide/siRNA complexes at different N/P ratios using scrambled siRNA. Heparin sulfate dissociated siRNA from the peptide/siRNA complexes (FBS 25%, incubation time of 24 h). The band representing negative control (scrambled siRNA exposed to saline for 24 h at 37 °C) was quantified as 100%. The bands were quantified using Image Lab software version 6.0.1.

The acquired results provided information on the ability of oleyl-conjugated peptides to shield siRNA from enzymatic degradation when FBS was present. While unmodified (WRH)_1_ was inefficient at all N/P ratios (Figure 5), all the other studied peptides showed a comparable efficiency for the protection of siRNA from enzymatic degradation at an N/P ratio ≥ 5 (Figure 5). The data indicate the potential of these peptides as protective carriers for effective siRNA delivery, as shown by analyzing siRNA stability inside the complex following exposure to FBS. The peptides with a higher number of R residues presumably have strong binding with siRNA, and protect siRNA against enzymatic degradation, especially at higher N/P ratios. The results from this study further validate the data from previous reports on oleyl-conjugated peptides [13,15,17,18].

### 2.5. In Vitro Cytotoxicity of Peptide/siRNA Complexes

**Figure 6:**
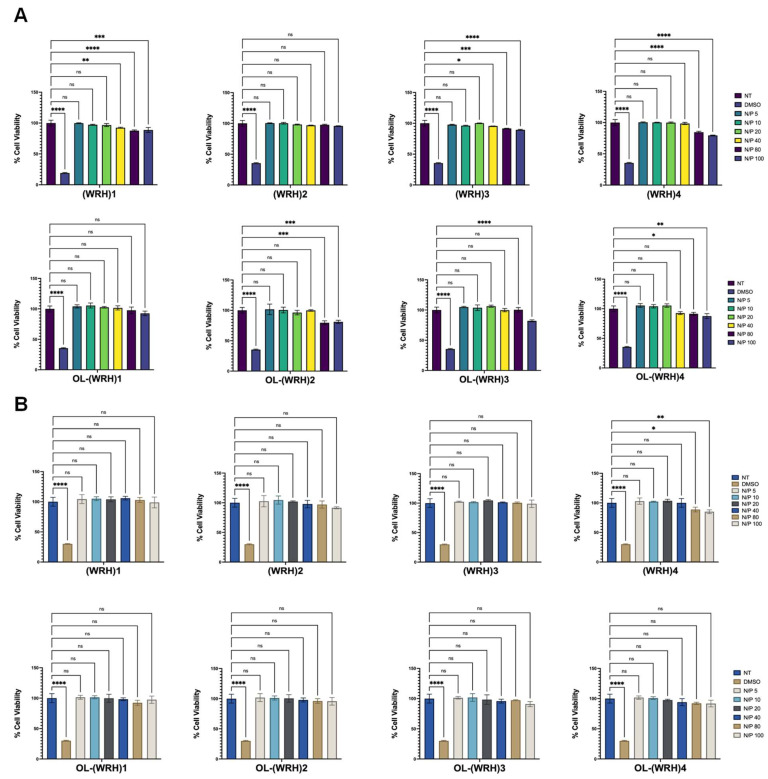
Cytotoxicity of peptide/siRNA complexes in (**A**) MDA-MB-231 and (**B**) MCF-7 cells. NT represents the non-treatment group, which serves as the negative control, whereas 35% *v*/*v* DMSO was used as the positive control. Graphs represent mean normalized values (n = 3). Error bars indicate standard deviation; ns = non-significant (*p*-value > 0.05), * *p*-value < 0.05, ** *p*-value < 0.001, *** *p*-value < 0.0005, and **** *p*-value < 0.0001.

**Figure 7:**
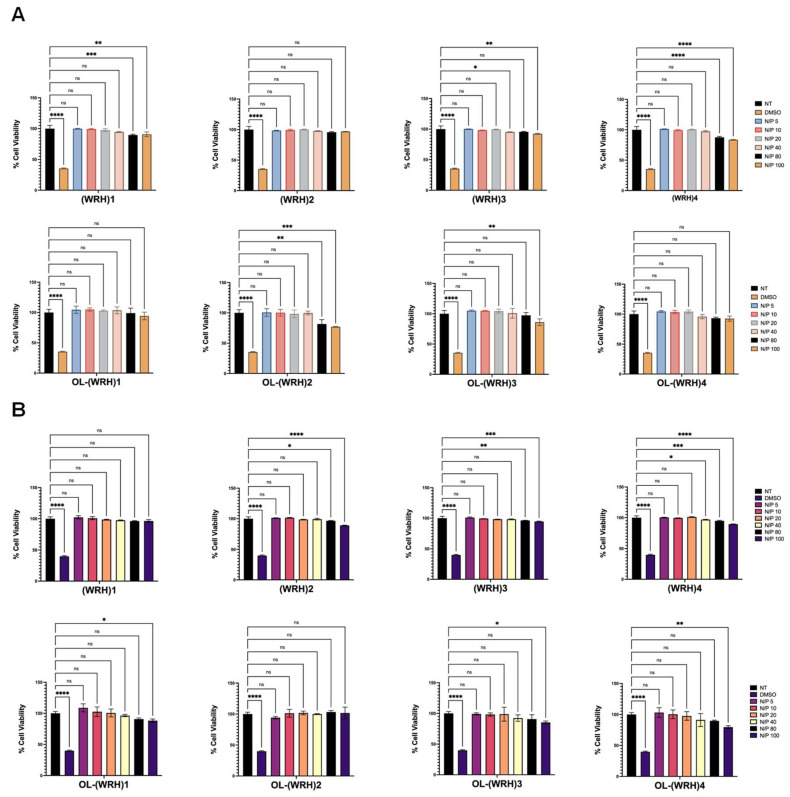
Cytotoxicity of peptide/siRNA complexes in (**A**) SK-OV-3 and (**B**) HEK-293 cells. Graphs represent mean normalized values (based on no treatment or the NT group, n = 3). Error bars indicate the standard deviation; ns = non-significant (*p*-value > 0.05), * *p*-value < 0.05, ** *p*-value < 0.001, *** *p*-value < 0.0005, and **** *p*-value < 0.0001.

This study aimed to assess these complexes’ potential as a delivery system for siRNA-mediated gene silencing in cancer cells. Our initial experiments involved the use of the MDA-MB-231 (Figure 6A), MCF-7 (Figure 6B), SK-OV-3 (Figure 7A), and HEK-293 (Figure 7B) cell lines for cell culture and in vitro cytotoxicity studies. After 72 h of incubation, we used an MTS test to determine the cytotoxicity of the developed peptides complexed with scrambled siRNA. The peptide/siRNA complexes were applied to the cells at various concentrations and N/P ratios (from 10 to 100). The non-treated group (NT) served as the negative control, while 35% *v*/*v* DMSO was used as the positive control, which proved to be cytotoxic to over 70% of the cells. This finding corroborates the results from our previous reports [13,18].

The outcomes demonstrated that the peptide/siRNA complexes displayed variable degrees of cytotoxicity depending on the cell line and the N/P ratio utilized. The cytotoxicity data are represented below in Figure 6. The statistical analysis of the data demonstrated that all peptide/siRNA complexes showed no significant cytotoxicity as compared with non-treated cells in all the cell lines up to an N/P ratio of 80. However, with an increase in the N/P ratio, the complexes showed dose-dependent cytotoxicity in all the above-mentioned cell lines. The significance of toxicity for each individual treatment group was assessed via statistical analysis and is represented by the *p*-values in Figure 6 and Figure 7. Therefore, an N/P ratio of 40 was selected as a safer ratio of peptide/siRNA complexes for the remaining cell assays to ensure the cells were viable during the experiments.

### 2.6. Cellular Internalization of siRNA Using Flow Cytometry

**Figure 8:**
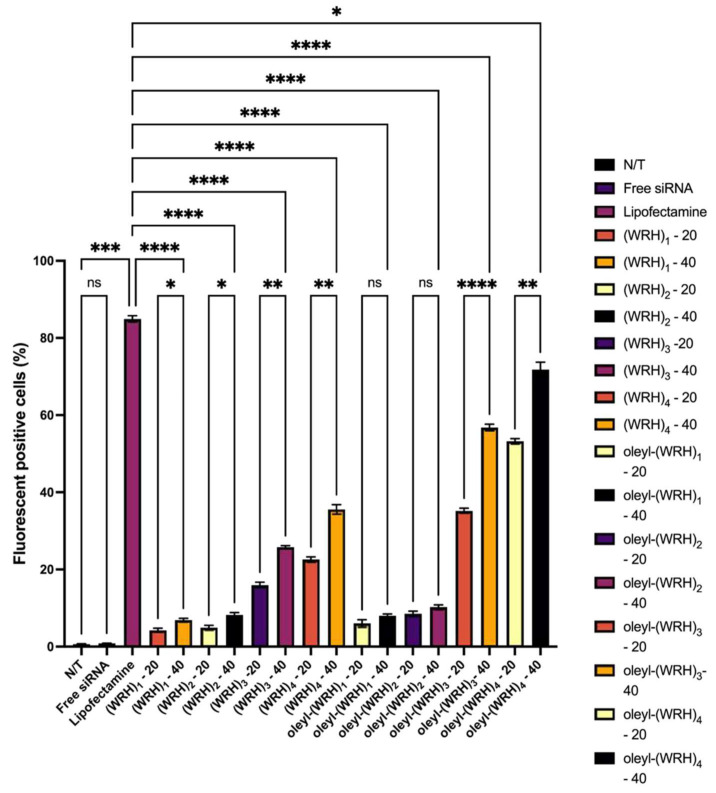
Flow cytometry of non-oleyl- and oleyl-conjugated-siRNA complexes at N/P ratios of 20 and 40 in MDA-MB-231 cells using Alexa Fluor 488-labeled scrambled siRNA after 24 h incubation. Bars represent the mean value (*n* = 3), and the error bars represent standard deviations; ns = non-significant (*p*-value *>* 0.05), * *p*-value < 0.05, ** *p*-value < 0.001, *** *p*-value < 0.0005, and **** *p*-value < 0.0001.

**Figure 9:**
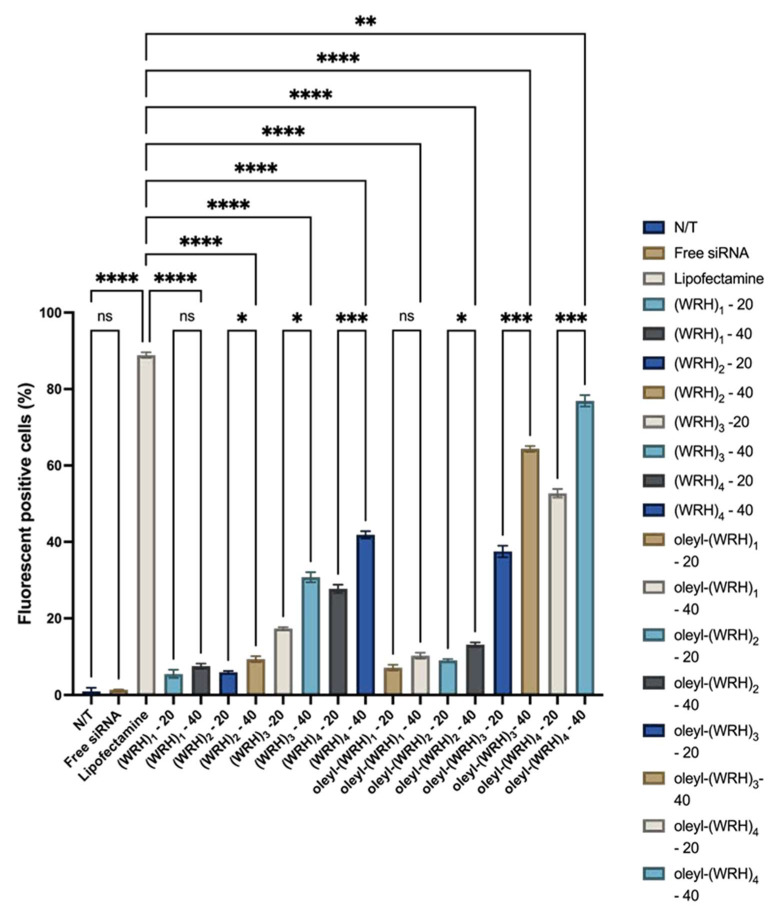
Flow cytometry of non-oleyl- and oleyl-conjugated siRNA complexes at N/P ratios of 20 and 40 in SK-OV-3 cells using Alexa Fluor 488-labeled scrambled siRNA after 24 h incubation. Bars represent the mean value (*n* = 3), and the error bars represent standard deviations; ns = non-significant (*p*-value > 0.05), * *p*-value < 0.05, ** *p*-value < 0.001, *** *p*-value < 0.0005, and **** *p*-value < 0.0001.

Based on the binding affinity, stability, and gel electrophoresis assays, the peptides were examined for the cellular delivery of siRNA. We performed a flow cytometry experiment to examine the cellular uptake of non-oleyl- and oleyl-conjugated peptide/siRNA complexes by monitoring the Alexa Fluor 488-labeled scrambled siRNA. This study was carried out on the cell lines MDA-MB-231 and SK-OV-3 (Figure 8 and Figure 9). After being exposed to the complexes for 24 h, the cells were trypsinized and thoroughly washed with a buffer solution to avoid non-specific binding and autofluorescence of the complexes with the cell membranes. After washing, the cells were subjected to flow cytometry analysis (see Section 4.8). The non-treated and siRNA-only treated cells served as negative controls. The gates of the FACS flow cytometer were set to reflect negligible fluorescent positive cells (<1%). Using the same settings, lipofectamine-treated cells, which showed >80% fluorescent-positive cells compared with non-treated cells, served as the positive control. The same gate settings were used to analyze the quantitative fluorescent-positive cells treated with peptide/siRNA complexes. The flow cytometry analysis provided quantitative evidence for the cellular internalization of siRNA up to ~60% and ~75% using oleyl-(WRH)_3_ and oleyl-(WRH)_4_, respectively, at N/P ratios of 40 after 24 h of incubation, in both MDA-MB-231 and SK-OV-3 cells (Figure 8 and Figure 9). These percentage internalization values reflect the quantitative amount of siRNA delivered inside cells compared with siRNA alone. A significant increase in mean fluorescent intensity and the siRNA uptake by the cells with a higher number of R residues was observed, where oleyl-(WRH)_4_ demonstrated the highest cellular internalization when compared with other peptides, presumably because of the stronger interactions of these peptides with negatively charged siRNA and a cell membrane phospholipid bilayer. Furthermore, the addition of the oleyl fatty acyl chain enhanced the siRNA delivery, possibly due to higher interactions with hydrophobic residues in the cell membrane. A comparable difference in the cellular uptake of lipofectamine and oleyl-(WRH)_4_ was observed. These results imply that the oleyl-conjugated peptides efficiently promoted siRNA uptake into the cancer cells. Furthermore, the data from this study validate the cellular uptake findings from previous reports by our group on oleyl-conjugated peptides for intracellular siRNA internalization [13,15,17,18].

### 2.7. Cellular Internalization Study of siRNA Using Confocal Microscopy

**Figure 10:**
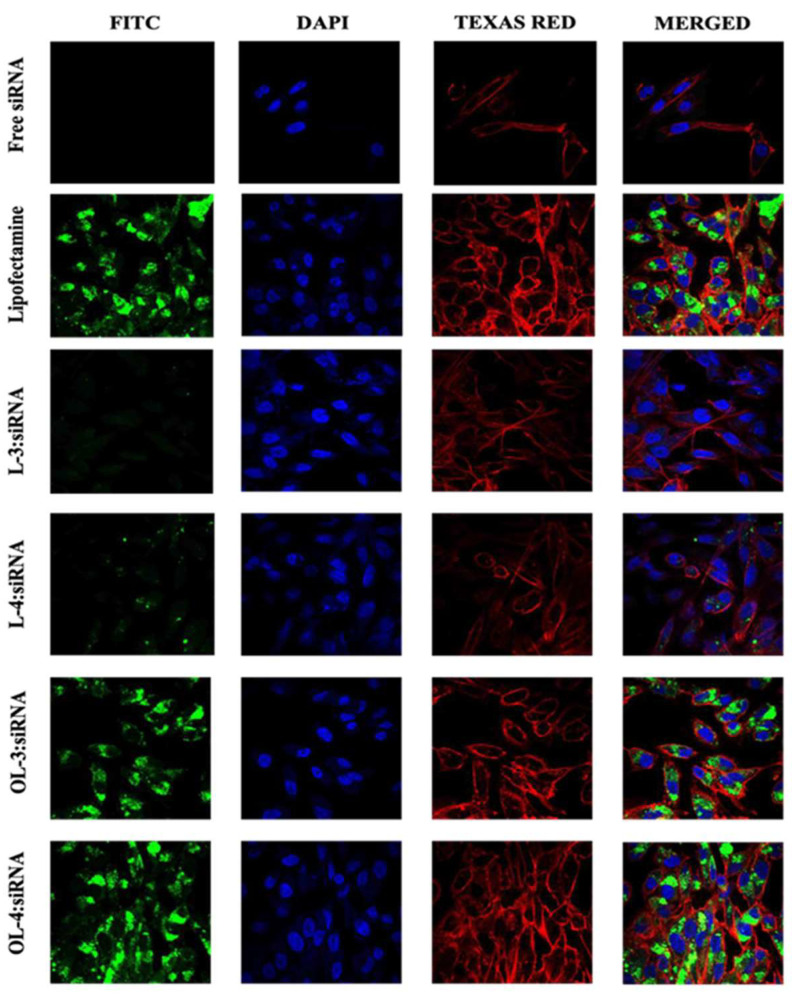
Confocal microscopy images of MDA-MB-231 cells treated with (WRH)_3_, (WRH)_4_, oleyl-(WRH)_3_, and oleyl-(WRH)_4_ complexes with Alexa Fluor 488-labeled scrambled siRNA after 24 h incubation. The cell membrane cytoskeleton of the cells was stained using Texas Red (TR) phalloidin (red color), while 4′,6-diamidino-2-phenylindole (DAPI) (blue color) was used to stain the nuclei of the cells.

**Figure 11:**
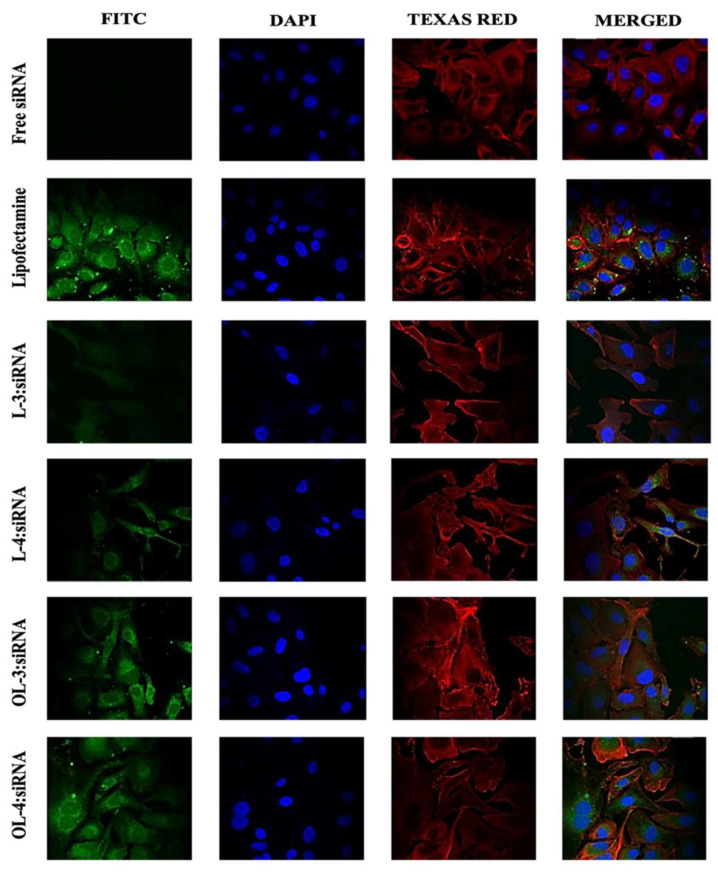
Confocal microscopy images of SK-OV-3 cells treated with (WRH)_3_, (WRH)_4_, oleyl- (WRH)_3_, and oleyl-(WRH)_4_ complexes with Alexa Fluor 488-labeled scrambled siRNA after 24 h incubation. The cell membrane cytoskeleton of the cells was stained using Texas Red (TR) phalloidin (red color), while DAPI (blue color) was used to stain the nuclei of the cells.

A siRNA uptake investigation utilizing confocal microscopy was conducted to corroborate the flow cytometry data. We performed microscopy in the cells according to the previously reported procedure [20]. Based on the binding affinity, stability, gel electrophoresis, and flow cytometry assays, oleyl-(WRH)_3_ and oleyl-(WRH)_4_ were selected for examining the cellular delivery of siRNA using confocal microscopy compared with non-oleyl (WRH)_3_ and (WRH)_4_. After treatment, rigorous washing of cells was performed to ensure the complete elimination of any peptide/siRNA complexes on the cell surface before fixing the cells and subsequent staining to visualize the cellular uptake of siRNA in the cells. Figure 10 and Figure 11 demonstrate the intracellular delivery of siRNA by cells using the peptides oleyl-(WRH)_3_ and oleyl-(WRH)_4_ at an N/P ratio of 40. The cellular uptake for lipofectamine, oleyl-(WRH)_3_, and oleyl-(WRH)_4_ was more significant in MDA-MB-231 cells than in SK-OV-3 cells. Both oleyl-(WRH)_3_ and oleyl-(WRH)_4_ exhibited cytosolic delivery. These data indicate that the peptides are promising siRNA delivery agents for both breast and ovarian cancer cells. These data were consistent with flow cytometry data.

### 2.8. STAT-3 Silencing Using Peptide:siRNA Complexesin MDA-MB 231 and SK-OV-3 Cells

STAT-3 exhibits a notable overexpression in several cancer types, including breast and ovarian cancer. Thus, it was chosen as the model protein [13]. By examining the expression of STAT-3 in peptide/siRNA complex-treated cells, the silencing efficiency of STAT-3 siRNA delivered by oleyl-conjugated peptides was determined. Peptide/siRNA complexes were treated with MDA-MB-231 and SK-OV-3 cells for 48 h at a final siRNA concentration of 50 nM. Protein concentration was established after preparing cell lysates.

**Figure 12:**
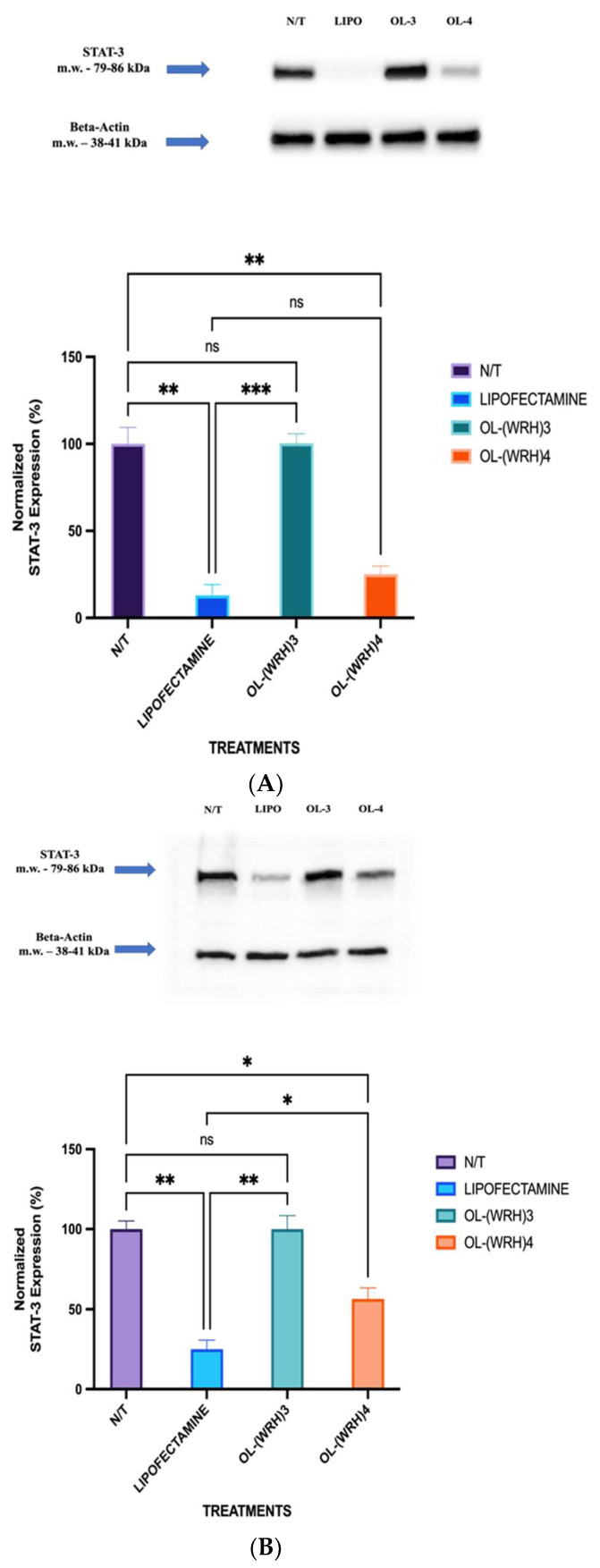
Comparison of STAT-3 protein downregulation efficiencies of the oleyl-conjugated peptides oleyl-(WRH)_3_ and oleyl-(WRH)_4_ at a N/P ratio of 40 in (**A**) MDA-MB-231 cells and (**B**) SK-OV3 cells. Error bars indicate the standard deviation (*n* = 3); ns = non-significant (*p*-value > 0.05), * *p*-value < 0.05, ** *p*-value < 0.001, and *** *p*-value < 0.0005.

Compared with the non-treated cells, results showed that STAT-3 expression was significantly lower in those treated with oleyl-(WRH)_4_-siRNA complexes. Considering that the expression of the STAT-3 gene in non-treated cells is 100%, the commercially available Lipofectamine 2000 showed ~87% silencing of STAT3. Oleyl-(WRH)_3_ showed no silencing, while a ~75% downregulation of STAT3 was observed in the presence of oleyl-(WRH)_4_ in MDA-MB-231 cells (Figure 12A).

Furthermore, to evaluate and confirm the gene silencing efficiency of the oleyl-conjugated peptides in a different cell line, the peptides were targeted and evaluated using ovarian cancer (SK-OV-3) cells. The positive control for the experiment Lipofectamine 2000 silenced the STAT3 gene by ~75%. The oleyl-conjugated peptide oleyl-(WRH)_3_ showed no silencing, but oleyl-(WRH)_4_ successfully exhibited ~45% silencing of STAT-3 in SK-OV-3 cells (Figure 12B). These results demonstrate that the oleyl-conjugated peptide/siRNA complexes were successfully delivered into the cells, where they specifically downregulated the target protein STAT-3 in MDA-MB-231 and SKOV-3 cell lines. The variability of the STAT-3 silencing across different cell lines may be attributed to differences in cell membrane composition and STAT-3 expression level in these cell lines. These findings corroborate our previous reports [13,18]. While oleyl-(WRH)4 was less effective than lipofectamine in inducing silencing effects, its non-cytotoxic nature could be advantageous for this application. Moreover, this peptide can be potentially optimized through structural modifications to enhance its silencing effectiveness.

## 3. Conclusions

In this study, we synthesized and characterized linear peptides with W, R, and H residues and fatty-acylated them using oleic acid on solid-phase peptide synthesis. To facilitate siRNA delivery, peptides were mixed with scrambled siRNA, which formed stable nano-complexes with hydrodynamic diameters below 79 nm and positive zeta potentials. Analyzed by dynamic light scattering, these complexes exhibited positive zeta potentials (13–18 mV) and sizes ranging from 58 to 79 nm, indicating a desirable size distribution. Gel electrophoresis assays confirmed 100% siRNA binding at an N/P ratio of 40. Peptide/siRNA complexes, particularly oleyl-conjugated peptides, demonstrated resistance to enzymatic degradation. Cytotoxicity studies on various cancer cell lines revealed minimal cytotoxicity at an N/P ratio ≤ 80, with efficient cellular uptake observed in MDA-MB-231 and SK-OV-3 cells. Confocal microscopy confirmed cellular uptake, and the oleyl-(WRH)_4_ complex effectively downregulated STAT-3 expression in both cell lines, showcasing its successful gene-silencing capabilities. Furthermore, the Western blotting data clearly demonstrate the efficacy of the designed oleyl-conjugated peptides in silencing the target gene STAT-3 across different cell lines. This effectiveness suggests their potential for investigating in vivo gene silencing for cancer and infectious disease targets. While commercially available lipofectamine shows remarkable gene silencing, it comes with significant cytotoxicity and is unsuitable for in vivo use. In contrast, the designed peptides in this study exhibit adequate safety in cancer cell lines at concentrations significantly higher than those used in animal models, making them suitable for further animal studies. These findings highlight the potential of oleyl-conjugated peptides as effective siRNA delivery vehicles for targeted gene silencing in cancer cells, warranting further optimization and in vivo exploration.

## 4. Materials and Methods

### 4.1. Materials

The amino acid building blocks Fmoc-His(Trt)-OH, Fmoc-Arg(Pbf)-OH, and Fmoc-Trp(Boc)-OH were obtained from AAPPTec LLC (Louisville, KY, USA). The resin H-His-(Trt)-Cl-2-chlorotrityl (loading capacity = 0.724 mmol/g) was purchased from Sigma-Aldrich (St. Louis, MO, USA). Coupling reagents, including HCTU, DMF, DIPEA, trifluoroacetic acid (TFA), acetic acid, triisopropylsilane (TIS), and piperidine, were purchased from Sigma-Aldrich Chemical Co. (Milwaukee, WI, USA) and used without further purification. F′-siRNA (Catalogue No. AM4635-AM4636) was obtained from Ambion Inc. (Austin, TX, USA). All cell biology reagents were procured from Wilken Scientific (Pawtucket, RI, USA) or Fisher Scientific (Hanover Park, IL, USA).

The chemical structures of the peptides were confirmed by analyzing their masses using a high-resolution matrix-assisted laser desorption/ionization-time-of-flight (MALDI-TOF) mass spectrometer (model # GT 0264, Bruker Inc., Fremont, CA, USA) in positive ion mode with α-cyano-4-hydroxycinnamic acid as the matrix. Crude peptide purification was carried out using a reverse-phase high-performance liquid chromatography (RP-HPLC) system (Shimadzu LC-20AP, Canby, OR, USA) equipped with a Waters XBridge BEH 130 preparative column (10 µm, 110 Å, 21.2 × 250 mm), employing a gradient elution of acetonitrile and water with 0.1% trifluoroacetic acid (TFA, *v*/*v*) at a flow rate of 6 mL/min, and monitored at 220 nm.

Hanks’ Balanced Salt Solution (HBSS), fetal bovine serum (FBS), penicillin (10,000 U/mL), and streptomycin (10 mg/mL) were supplied by Life Technologies (Grand Island, NY, USA). Western blot materials, including 10% Mini-PROTEAN^®^ TGX Stain-Free Protein Gels, Clarity Western ECL Substrate, Trans-blot^®^ Turbo TM Cassettes, and Trans-Blot^®^ Turbo Mini PVDF Transfer Packs, were obtained from Bio-Rad (Hercules, CA, USA). 4,6-Diamidino-2-phenylindole (DAPI) was provided by Vector Laboratories (Burlingame, CA, USA). Phosphate-buffered saline (PBS, 20× concentration), sulforhodamine 101 (Texas Red), and other chemicals were purchased from VWR (Radnor, PA, USA). Scrambled negative control siRNA (Catalogue No. AM4635), Alexa Fluor 488-labeled negative control siRNA (Catalogue No. 1027292), and siRNA targeting STAT3 (Catalogue No. SI02662338) were acquired from Life Technologies (Grand Island, NY, USA). Monoclonal antibodies against STAT3 (Mouse, Catalogue No. 9139S) and GAPDH (Mouse, Catalogue No. 97166S) were supplied by Cell Signaling Technology, Inc. (Danvers, MA, USA). Mouse IgG HRP-conjugated antibody was purchased from R&D Systems (Catalogue No. HAF007).

### 4.2. Solid-Phase Peptide Synthesis of Unmodified and Oleyl-Conjugated Peptides

The peptides were synthesized via Fmoc/tBu solid-phase peptide synthesis, following the procedures detailed in previously reported methods [18], exemplified in Scheme 1. In summary, the synthesis utilized preloaded H-His-(Trt)-Cl-2-chlorotrityl resin (loading capacity = 0.724 mmol/g) as solid support. Peptides containing alternating tryptophan, arginine, and histidine residues were synthesized on a 0.50 mmol scale, following the specified peptide sequence. Subsequently, complete cleavage was achieved by incubating with a freshly prepared cleavage cocktail reagent R (trifluoroacetic acid (TFA): thioanisole: anisole: dithiothreitol (DTT) (92:5:2:3; *v*/*v*/*v*/*v*)) for 4 h to obtain non-oleyl peptides.

For the oleyl-conjugated peptides, following the conjugation of the last amino acid and Fmoc deprotection steps, *N*-terminal acylation with oleic acid was carried out in the presence of HCTU/DIPEA. The resin was thoroughly washed with DMF. Fmoc deprotection was achieved using piperidine (20% *v*/*v*) in DMF, and the Kaiser test confirmed the completion of the coupling process with all free amino groups reacted. Finally, all peptide conjugates were cleaved using a freshly prepared cleavage cocktail reagent R (TFA: thioanisole: anisole: DTT, 92:5:2:3; *v*/*v*/*v*/*v*) for 4 h.

All crude peptides were precipitated using cold diethyl ether, centrifuged, and subsequently purified using reverse-phase high-performance liquid chromatography (RP-HPLC) on a Shimadzu LC-20AP Prominence system. The purification utilized a gradient elution system consisting of water with 0.1% TFA and acetonitrile (CH_3_CN) with 0.1% TFA (*v*/*v*) (5% to 95% CH_3_CN over 60 min) on a C18 column (00G-4436-P0-AX, Gemini Prep C18, 10 μm particle size). Fractions were collected and analyzed using MALDI-TOF mass spectrometry. Fractions containing the expected compounds were pooled and lyophilized to obtain solid peptide powders. Analytical HPLC was conducted to confirm the purity (>95%) of the most effective compounds used in biological assays. Table 1 outlines the manual peptide synthesis steps. Supplementary material provides spectrum of crude MALDI-TOF data and purity of peptides used in the biological assays (Figures S1–S12). 

(WRH)_1_: MALDI-TOF (*m*/*z*) C_23_H_33_N_9_O_4_ Calculated: 499.2645, Found: 499.4015 [M+2H]^+^; (WRH)_2_: MALDI-TOF (*m*/*z*) C_46_H_62_N_18_O_7_ Calculated: 978.5038, Found: 978.4187 [M+2H]^+^; (WRH)_3_: MALDI-TOF (*m*/*z*) C_69_H_90_N_27_O_10_ Calculated: 1456.7358, Found: 1456.5664 [M+H]^+^; (WRH)_4_: MALDI-TOF (*m*/*z*) C_92_H_119_N_36_O_13_ Calculated: 1935.9752, Found: 1935.6767 [M+H]^+^; Oleyl-(WRH)_1_: MALDI-TOF (*m*/*z*) C_41_H_65_N_9_O_5_ Calculated: 763.5098, Found: 763.6974 [M+2H]^+^; Oleyl-(WRH)_2_: MALDI-TOF (*m*/*z*) C_64_H_94_N_18_O_8_ Calculated: 1242.7491, Found: 1242.9278 [M+2H]^+^; Oleyl-(WRH)_3_: MALDI-TOF (*m*/*z*) C_87_H_122_N_27_O_11_ Calculated: 1720.9812, Found: 1720.6871 [M+H]^+^; Oleyl-(WRH)_4_: MALDI-TOF (*m*/*z*) C_112_H_152_N_36_O_14_ Calculated: 2201.2278, Found: 2201.7647 [M+2H]^+^.

### 4.3. Complex Formation of Oleyl-Conjugated Peptides and Scrambled siRNA

The complexation of oleyl-conjugated peptides and siRNA involved physically mixing them together. The stock solution of peptides (1 mM) was prepared by dissolving purified powdered peptide in sterile deionized water with rigorous vertexing. This solution was then serially diluted to obtain the desired concentrations for making complexes with siRNA. Given the small quantity of the peptide used, all synthesized conjugated peptides were effectively dissolved in purified deionized water with thorough shaking. The stock solution of siRNA [2000 nM] was prepared according to the manufacturer’s instructions and was serially diluted to achieve the desired concentrations for complex formation. Appropriate volumes of oleyl-conjugated peptides and scrambled siRNA solutions were combined in Hanks’ Balanced Salt Solution (HBSS) buffer for 30 min at room temperature (25 °C) to form the complexes, as previously described by our group [13,18]. The detailed protocol for the calculation is provided in a report by Grayson et al. [21]. These complexes were formed through ionic interactions between the positively charged arginine residues of the conjugates and the negatively charged phosphate groups of siRNAs.

Several methods for describing the concentration of “carrier (acylated peptides) and nucleic acid (siRNA) complexes” have been reported in the literature, including N/P ratio, weight/weight ratio, and molar ratio. The quantities of siRNA and peptide used in this study and the previous studies from our group are in nanomoles and micromoles, respectively [14,17,18]. We chose N/P ratio as a convenient way of describing the quantities of siRNA and the carrier peptides. N/P ratio describes the ratio of the quantity of the carrier peptide to the siRNA, where N represents the moles of ionizable nitrogen in the delivery agent and P represents the moles of phosphate groups in siRNA. It was calculated using the following formula:

N/P = (no. of moles of peptide) × (no. of ionized nitrogen atoms)/(no. of moles in siRNA) × 48

The final concentration of siRNA complexes remained constant, while the concentration of the conjugates was incrementally adjusted to achieve various N/P ratios.

### 4.4. Dynamic Light Scattering

The peptide/siRNA complexes were analyzed using dynamic light scattering (DLS) methodology. A Malvern Nano ZS Zetasizer (Westborough, MA, USA) was employed to measure the hydrodynamic diameter and surface charge of the complexes at 25 °C. Zeta potential measurements were conducted at 40 V using disposable folded capillary cells (DTS1070). The instrument was calibrated with a transfer standard, and disposable cuvettes were utilized for determining the hydrodynamic diameter.

A specific N/P ratio was selected for the DLS experiments, maintaining a final siRNA concentration of 50 nM within the complexes. The complexes were freshly prepared and analyzed for the DLS studies. In our previous studies, we prepared the complexes in advance and froze them at −20 °C for 72 h, which did not affect the reproducibility of DLS results (data not published). Each measurement was performed in triplicate, with each run consisting of 20 scans. Zeta potential values were calculated using the Smoluchowski model, and all results met the instrument’s quality standards.

Particle size analysis was conducted at identical N/P ratios using consistent concentrations of siRNA and oleyl-conjugated peptides. Each sample was analyzed in triplicate with an automatic attenuator setting. The results were validated against the instrument’s quality standards and reported as the mean ± SD from three independent experiments.

### 4.5. Gel Shifting Assay

Gel electrophoresis was employed to assess the binding affinity of the peptides with scrambled siRNA. The synthesized peptides were mixed with siRNA at N/P ratios ranging from 0 to 60, maintaining a final siRNA concentration of 36 nM in the complexes. Complex formation occurred in 1X PBS buffer at room temperature. The negative control (N/P ratio 0) lacked peptides entirely.

Freshly prepared 1% agarose gel was stained with 0.5 μg/mL ethidium bromide. Prior to loading into the wells, each complex received 5 μL of 6× purple gel loading dye. Gel electrophoresis was conducted at 70 V and 400 mA for 20 min in TAE buffer (2 M Tris base, 1 M glacial acetic acid, 0.5 M sodium EDTA, pH 8.3). Band visualization utilized the ChemiDoc XRS+ system with CCD high-resolution imaging (Bio-Rad Imager), and band intensities (indicative of unbound siRNA) were quantified using Image LabTM software. The experiment was performed in triplicate, and the percentage of binding (%Binding) was calculated using the following formula:

100% × [(control siRNA − free siRNA)/control siRNA].

### 4.6. Protection of siRNA against Enzymatic Degradation

In the harsh biological environment of the human body, various endonucleases rapidly degrade nucleic acids upon entry. Here, we investigated the ability of oleyl-conjugated peptides to protect siRNA from these endonucleases. Peptide/siRNA complexes were prepared at N/P ratios ranging from 0 to 60, with a final siRNA concentration of 36 nM. Fetal bovine serum (FBS) served as a positive control, while Hanks’ Balanced Salt Solution (HBSS) served as the negative control, representing 100% intact siRNA.

Each peptide/siRNA complex at different N/P ratios was exposed to a 25% (*v*/*v*) FBS solution in HBSS and then incubated at 37 °C for 24 h. After incubation, the heparin competition assay was employed to dissociate the peptide/siRNA complex and assess the amount of intact siRNA remaining after exposure to FBS. Specifically, 5 μL of a 2:3 mixture (*v*/*v*) of heparin (5% solution in normal saline) and ethylenediaminetetraacetic acid (0.5 mM) was added to each complex and incubated for 10 min. Subsequently, 5 μL of 6X purple gel loading dye was added to each mixture, and samples were analyzed using 1% ethidium bromide gel electrophoresis at 70 V and 400 mA for 20 min. Gels were visualized under UV illumination, and band intensities were quantified using Image LabTM software 6.0.1.

### 4.7. Cell Culture and in Vitro Cytotoxicity Assay

The cell lines selected for this study included MDA-MB-231 (ATCC No. HTB-26, triple-negative breast cancer cell line), MCF-7 (ATCC No. HTB-22, breast adenocarcinoma cells), HEK-293 (ATCC No. CRL-1573, normal kidney cells), and SK-OV-3 (ATCC No. HTB-77, human epithelial ovary adenocarcinoma cells).

MDA-MB-231, MCF-7, and SK-OV-3 cell lines were cultured in DMEM media supplemented with 10% fetal bovine serum (FBS 10%) and a penicillin–streptomycin solution (10,000 units of penicillin and 10 mg of streptomycin per mL in 0.9% NaCl, 1%). All cell lines were maintained in a humidified atmosphere containing 5% CO_2_ and 95% air at 37 °C. They were handled under sterile conditions within biological safety cabinets throughout the study.

The cytotoxicity of the synthesized oleyl-conjugated peptides complexed with scrambled siRNA was evaluated in vitro using the aforementioned cell lines. Peptide/siRNA complexes were prepared at N/P ratios ranging from 10 to 100.

For the assay, 10,000 cells per 0.1 mL were seeded into each well of a 96-well plate using a multichannel pipette and allowed to adhere for 24 h in a standard cell culture incubator. Cell health and confluency were assessed after the initial 24 h period. Subsequently, varying concentrations of freshly prepared peptide/siRNA complexes were added to each well in triplicate and incubated for 48 h at 37 °C in a humidified atmosphere with 5% CO_2_.

$$\%\;Cell\;viability=\left\{\frac{\left[(Average\;OD\;values\;of\;the\;samples\;treated\;with\;the\;compound)\;–\;(OD\;value\;of\;culture\;medium)\right]}{[(Average\;OD\;values\;of\;no\;treatment)-(OD\;value\;of\;culture\;medium)]\;}\right\}\times 100$$

### 4.8. Cellular Internalization of siRNA

The cellular uptake of oleyl-conjugated peptide/siRNA complexes was assessed using flow cytometry in live cells. Alexa Fluor 488-labeled scrambled siRNA was used to form complexes with oleyl-conjugated peptides. This investigation was conducted in MDA-MB-231 and SK-OV-3 cell lines.

Approximately 500,000 cells were seeded per well in a six-well plate and allowed to adhere for 24 h. Subsequently, cells were treated with conjugate-siRNA complexes and incubated for an additional 24 h. After treatment, cells were washed three times with 1X PBS, trypsinized, and centrifuged at 1000 rpm (96× *g*) for 5 min at 4 °C. The cell pellet was washed three more times with PBS to remove the residual medium. Finally, cells were resuspended in PBS for flow cytometric analysis using the Alexa Fluor 488 channel to quantify mean fluorescence intensity.

The mean fluorescence intensity in treated cells was calculated for each sample, referencing the signal calibration with non-treated cells serving as the negative control. Alexa Fluor 488-labeled scrambled siRNA acted as an additional negative control, while lipofectamine-siRNA-treated cells served as the positive control.

### 4.9. Cellular Uptake Study Using Confocal Microscopy

In light of previous studies demonstrating the use of fixed or live cells for imaging siRNA cellular uptake, confocal microscopy was employed to corroborate data acquired from flow cytometry. Approximately 400,000 MDA-MB-231 cells were seeded on coverslips in 6-well plates and allowed to adhere for 24 h. During this period, cell confluency and health were assessed under a standard microscope.

Peptide/siRNA complexes were prepared with a final siRNA concentration of 36 nM and administered to the cells. Non-treated (NT) cells and cells treated with siRNA alone (without carrier peptide) served as the negative controls, while lipofectamine/siRNA complexes were employed as the positive control. After 24 h of incubation with the complexes, cells on coverslips were washed three times with 1X PBS to remove non-specific binding and reduce autofluorescence from the complexes with the cell membranes. Following thorough washing, the cells were fixed with 3.7% formaldehyde solution for 30 min at room temperature.

Cell membranes were stained using Texas Red (TR) phalloidin Vector Laboratories (Burlingame, CA, USA) (1:250 in HBSS), and DAPI was used to stain cell nuclei. Images of fixed cells were captured using a Nikon A1R high-definition resonant scanning confocal microscope system equipped with a 60X objective and various filters for Alexa Fluor 488 (FITC), TR, and DAPI fluorescence channels using the NIS-Elements software (AR 4.30.02, 64 bit).

### 4.10. Protein Silencing Effect of siRNA (Western Blot)

STAT-3 was chosen as the model protein due to its significant overexpression in various types of cancer, including breast cancer. The aim of this study was to assess the silencing efficiency of STAT-3 siRNA delivered via oleyl-conjugated peptides by evaluating STAT-3 expression in cells treated with peptide/siRNA complexes. Non-treated cells served as the negative control, while cells treated with lipofectamine (20 μg/mL) complexed with STAT-3 siRNA (50 nM) served as the positive control.

To evaluate STAT-3 expression, Western blotting was performed. Approximately 500,000 MDA-MB-231 cells were seeded in T-25 flasks and incubated under standard growth conditions at 37 °C. After 24 h, cells were treated with peptide/siRNA complexes for 48 h, with a final siRNA concentration of 50 nM. Cell lysates were prepared using RIPA buffer according to standard protocols, followed by sonication in intervals to ensure complete cell lysis. The lysates were then centrifuged at 12,000 rpm (RCF = 13,870× *g*) at 4 °C for 15 min, and the supernatant was transferred to pre-cooled microtubes.

The total protein concentration in the lysates was determined using a BSA assay. Briefly, a working reagent (50:1 A: B) was added to standards and unknown samples in triplicate in a 96-well plate. After mixing and incubating for 30 min at 37 °C with 5% CO_2_, absorbance at 562 nm was measured using a SpectraMAX M5 microplate reader to quantify protein levels.

Approximately 15 μg of protein from each sample was loaded onto a 10% Mini-PROTEAN^®^ TGX Stain-Free protein gel, and electrophoresis was carried out in SDS buffer at 200 V for 30 min. The separated proteins were transferred to a Mini PVDF membrane using a Trans-Blot^®^ Turbo system. The membrane was blocked with 5% BSA for 3 h, followed by overnight incubation at 4 °C with a primary antibody (1:1000 in TBS-T). After washing the membrane with TBS-T, it was incubated with a secondary HRP-linked antibody (1:1000 in TBS-T) for 1 h, followed by additional washing steps.

Protein bands were visualized using an ECL Detect Kit and imaged with a ChemiDoc imager. Band intensities were quantified using Image LabTM software 6.0.1.

### 4.11. Statistical Analysis

Experiments were conducted in triplicate, and results are presented as mean ± standard deviation unless stated otherwise. Data from flow cytometry and Western blotting experiments were analyzed using standard one-way analysis of variance (ANOVA) with Tukey’s multiple comparison test. The effects of varying N/P ratios on particle size and zeta potential were evaluated using two-way ANOVA with Tukey’s multiple comparison test. A *p*-value > 0.05 was considered non-significant (ns). The statistical significance for all analyses was set at *p* < 0.05.

## Figures and Tables

**Table 1:** Optimization of manual peptide synthesis.

Step	Volume (μL)	Time (min)	Reagents	Reaction
1	15,000	30 (10 × 3)	DMF	Resin Swelling
2	25,000	120	Amino Acid 1 + DMF + HCTU + DIPEA	Coupling
3	15,000	15 (5 × 3)	DMF	Washing
4	10,000	30 (10 × 3)	20% Piperidine in DMF (*v*/*v*)	Deprotection
5	15,000	15 (5 × 3)	DMF	Washing
6	25,000	120	Amino Acid 2 + DMF + HCTU + DIPEA	Coupling
7	15,000	15 (5 × 3)	DMF	Washing
8	10,000	30 (10 × 3)	20% Piperidine in DMF (*v*/*v*)	Deprotection
9	15,000	15 (5 × 3)	DMF	Washing

Steps 3 to 9 were repeated until the desired sequence was obtained.

**Table 2:** Range of zeta potential values corresponding to the extent of stability.

Zeta Potential	Stability
±0–10 mV	Highly Unstable
±10–20 mV	Relatively Stable
±20–30 mV	Moderately Stable
±30 mV	Highly Stable

**Table 3:** Particle size analysis of peptide/siRNA complexes at an N/P ratio of 40.

Peptide/siRNA Complex at N/P 40	Z-Ave (d. nm)	PDI
(WRH)_1_	78.94 ± 1.3	0.213 ± 0.03
(WRH)_2_	71.43 ± 2.5	0.201 ± 0.06
(WRH)_3_	67.38 ± 1.6	0.217 ± 0.05
(WRH)_4_	61.84 ± 1.8	0.229 ± 0.03
oleyl-(WRH)_1_	75.66 ± 2.3	0.237 ± 0.07
oleyl-(WRH)_2_	69.76 ± 2.1	0.213 ± 0.04
oleyl-(WRH)_3_	64.28 ± 2.1	0.213 ± 0.04
oleyl-(WRH)_4_	58.74 ± 2.1	0.213 ± 0.04
Average	69.50 ± 1.10	

## Data Availability

Data is contained in the paper.

## Supplementary Materials

The following supporting information can be downloaded at https://www.mdpi.com/article/10.3390/ph17081083/s1, consisting of crude MALDI-TOF mass spectrometry spectra of synthesized peptides (Figures S1–S8) and purified analytical HPLC chromatograms (Figures S9–S12).
