# Supplementary material for: Design, Synthesis, and Evaluation of Oleyl-WRH Peptides for siRNA Delivery
Source: Pharmaceuticals (Basel). 2024 Aug 18;17(8):1083. doi: 10.3390/ph17081083 (PMC11357397; doi:10.3390/ph17081083)
Supplement: Supplementary file 1 [file pharmaceuticals-17-01083-s001.zip › pharmaceuticals-3121653-supplementary.pdf]

Supplementary Materials

Contents

1. MALDI-TOF mass spectrum of conjugated peptides Pages 1–8
2. Analytical HPLC chromatogram of conjugated peptides Pages 9-13

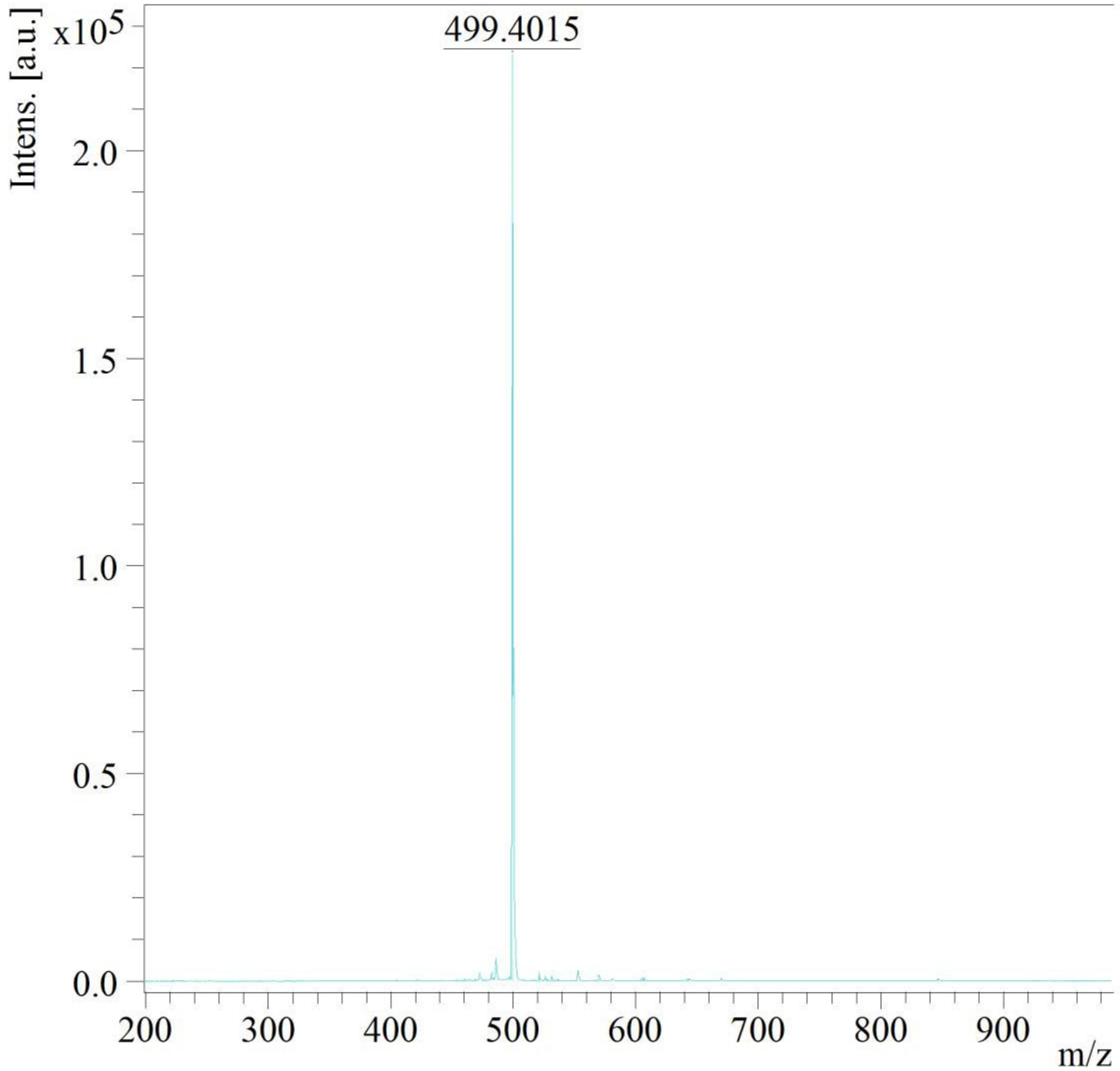

Figure S1. $(WRH)_1$: MALDI-TOF (m/z) $C_{23}H_{33}N_9O_4$ Calculated: 499.2645, Found: 499.4015 $[M+2H]^+$.

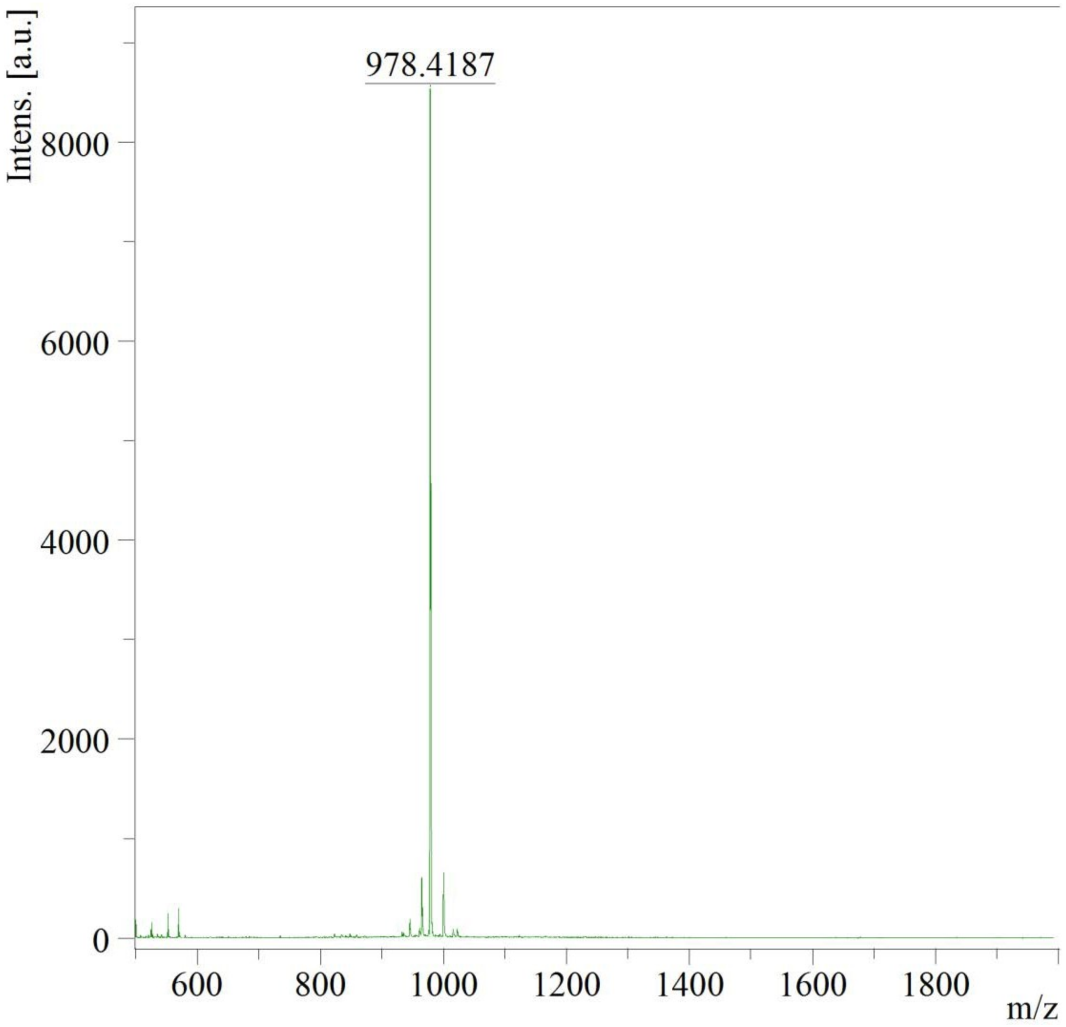

Figure S2. $(WRH)_2$: MALDI-TOF (m/z) $C_{46}H_{62}N_{18}O_7$ Calculated: 978.5038, Found: 978.4187 $[M+2H]^+$.

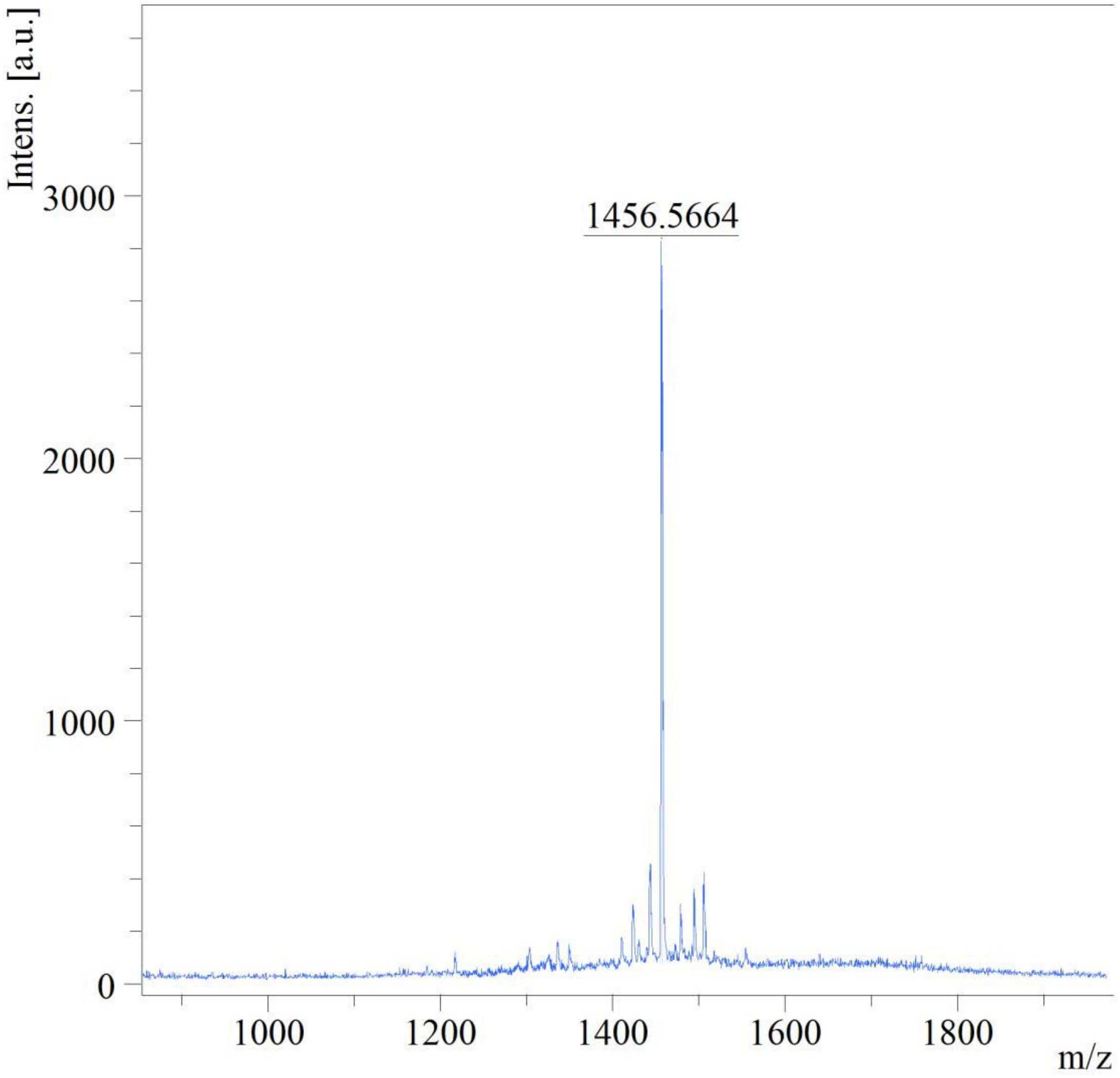

Figure S3. $(WRH)_3$: MALDI-TOF (m/z) $C_{69}H_{90}N_{27}O_{10}$ Calculated: 1456.7358, Found: 1456.5664 $[M+H]^+$.

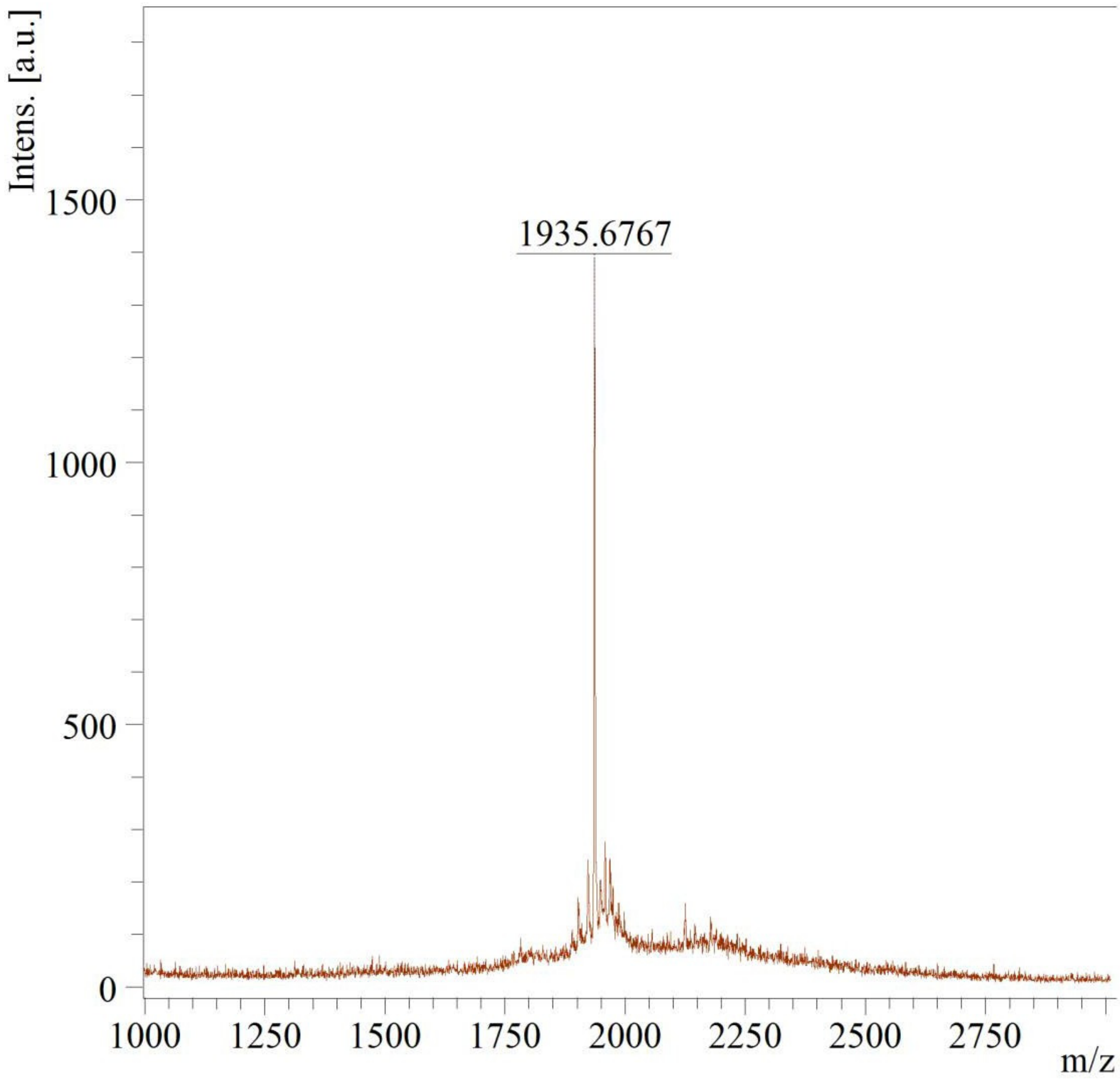

Figure S4. $(WRH)_4$: MALDI-TOF (m/z) $C_{92}H_{119}N_{36}O_{13}$ Calculated: 1935.9752, Found: 1935.6767 $[M+H]^+$.

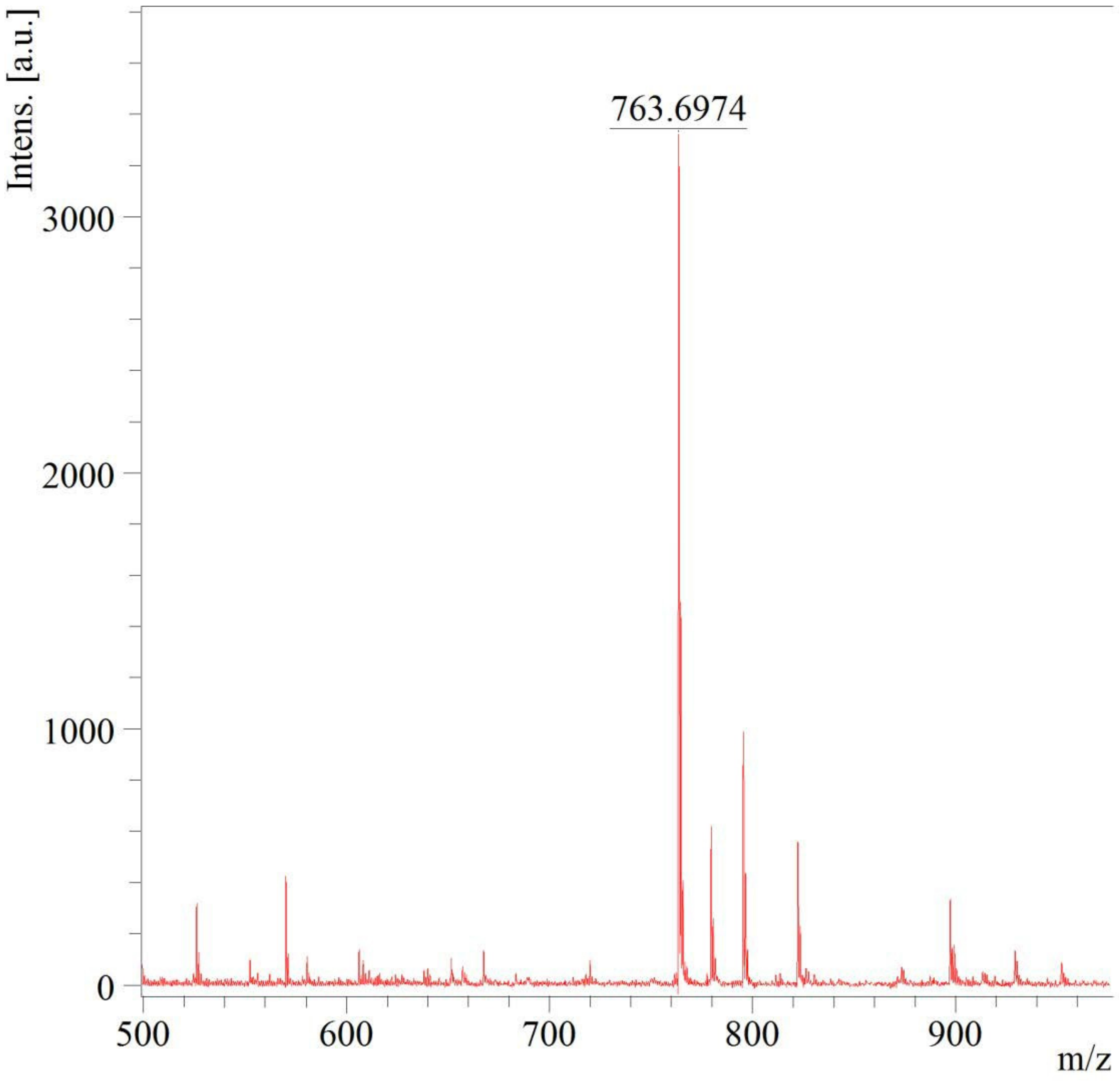

Figure S5. Oleyl-(WRH)$_1$: MALDI-TOF (m/z) $C_{41}H_{65}N_9O_5$ Calculated: 763.5098, Found: 763.6974 $[M+2H]^+$.

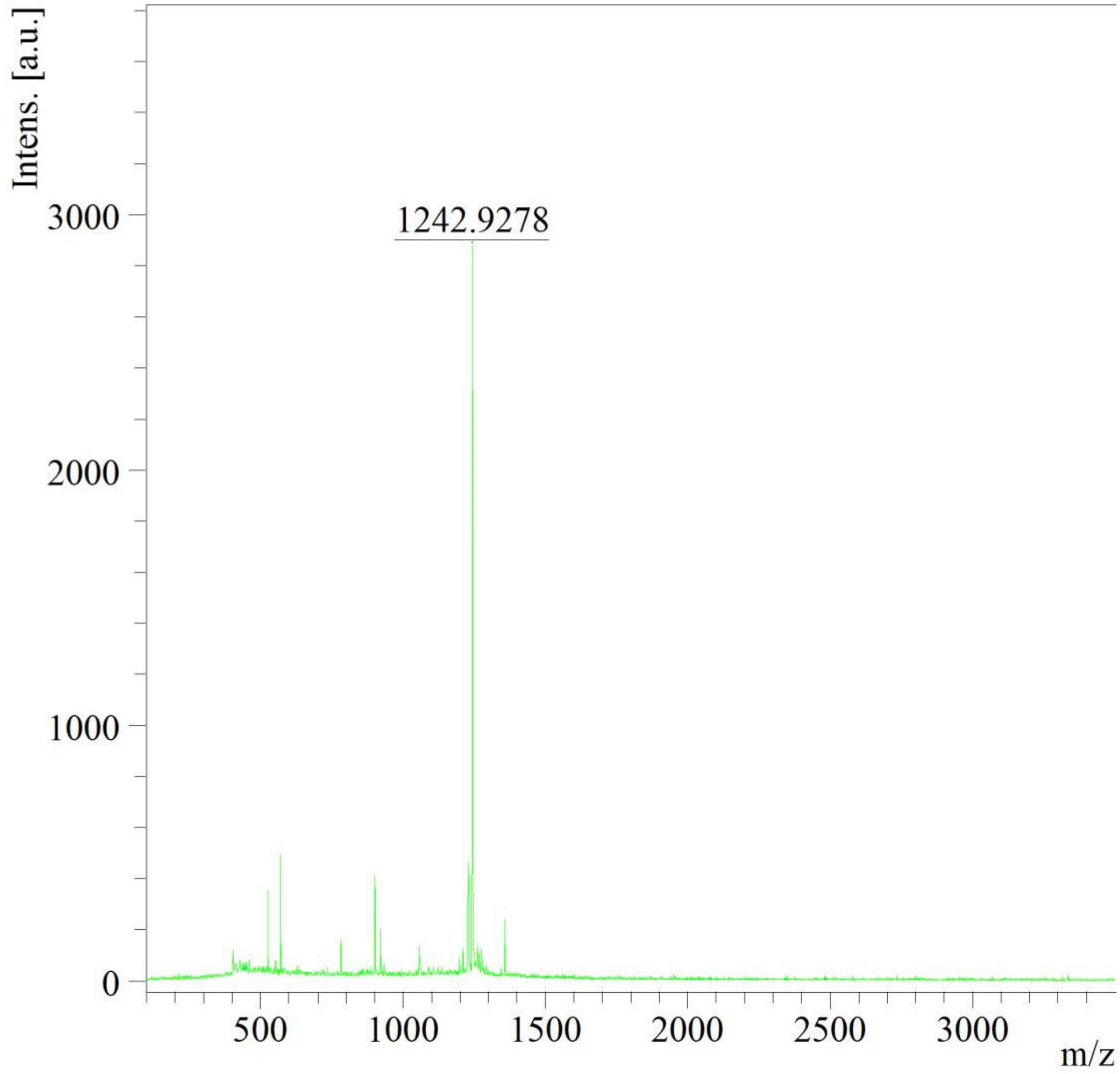

Figure S6. Oleyl-$(WRH)_2$: MALDI-TOF (m/z) $C_{64}H_{94}N_{18}O_8$ Calculated: 1242.7491, Found: 1242.9278 $[M+2H]^+$.

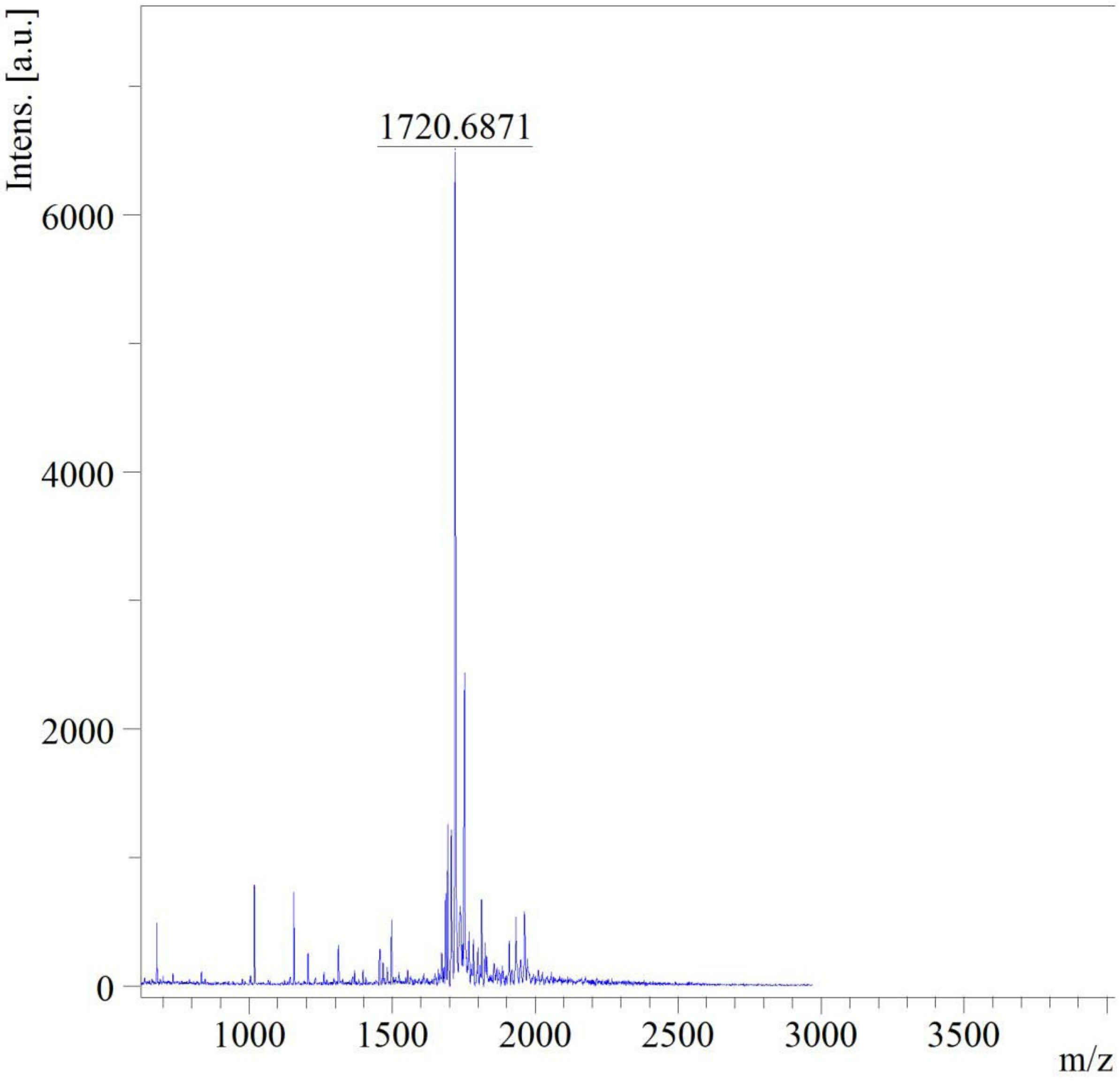

Figure S7. Oleyl-$(WRH)_3$: MALDI-TOF (m/z) $C_{87}H_{122}N_{27}O_{11}$ Calculated: 1720.9812, Found: 1720.6871 $[M+H]^+$.

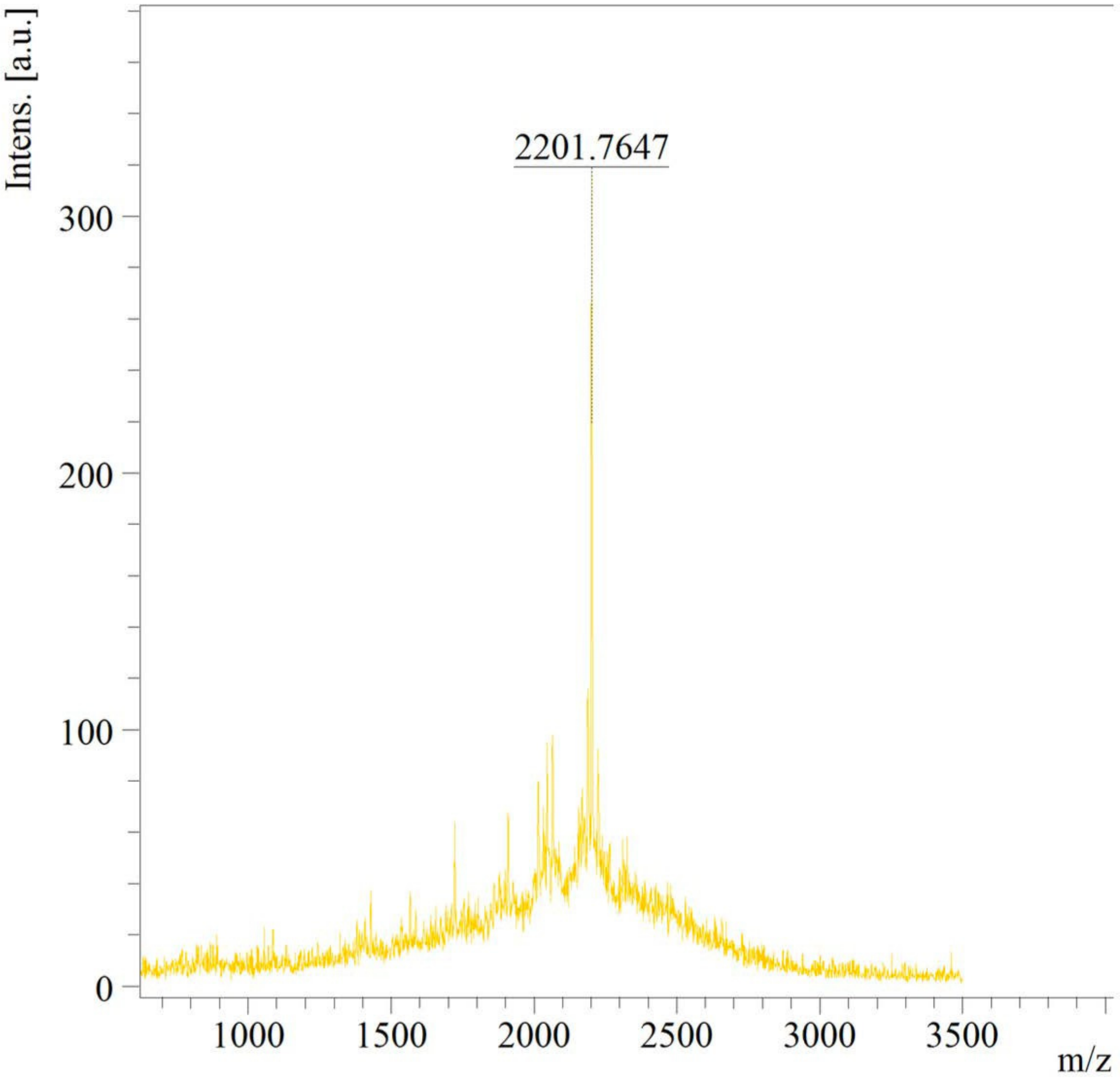

Figure S8. Oleyl-(WRH)4: MALDI-TOF (m/z) $C_{112}H_{152}N_{36}O_{14}$ Calculated: 2201.2278, Found: 2201.7647 $[M+2H]^+$.

Analytical HPLC Purification Analysis

The purity of the most efficient peptides $(WRH)_3$, $(WRH)_4$, oleyl-$(WRH)_3$, and oleyl-$(WRH)_4$ was determined using the reverse phase analytical HPLC method at a flow rate of 0.4 mL/min on Shimadzu (LC-20ADXR) with a gradient system of water with 0.1% Formic Acid (solvent A) and acetonitrile with 0.1% Formic Acid (solvent B), using (Phenomenex Luna, 4 um C18 150 × 4.6 mm HPLC Column).

**Time**	0.01	5.0	15.0	25.0	28.0	33.0	33.0	38.0
**% Solvent B**	5	5	20	70	95	95	5	5

HPLC CHROMATOGRAMS

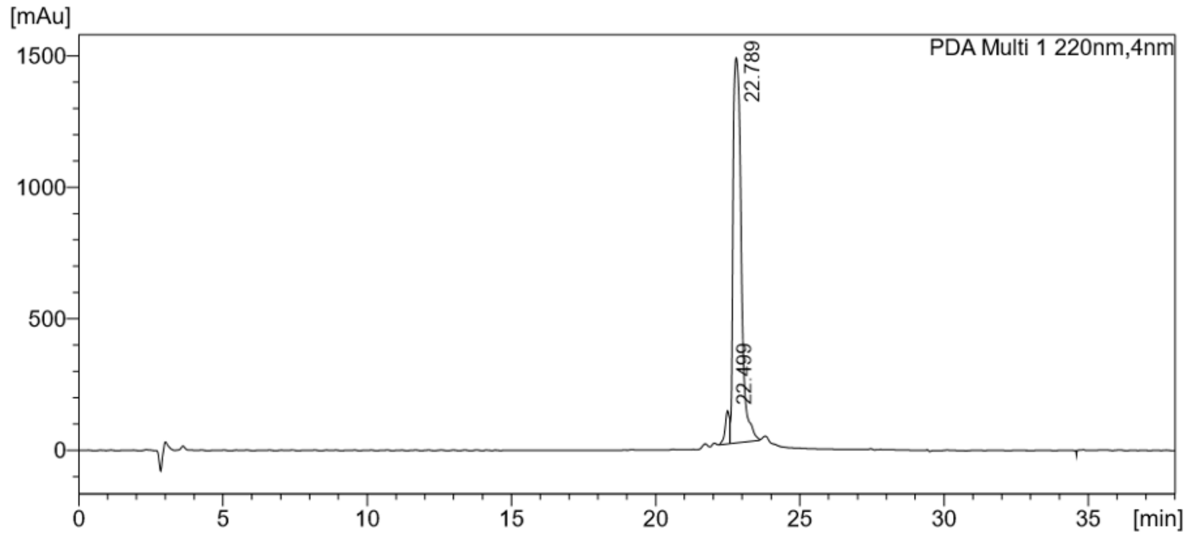

Figure S9. Analytical HPLC chromatogram of $(WRH)_3$

**Peptide**	$(WRH)_3$
**Pump A**	0.1% Formic Acid in 100% Water
**Pump B**	0.1% Formic Acid in 100% Acetonitrile
**Total Flow**	0.4 ml/min
**Retention Time**	22.789 min
**% Purity**	95.741

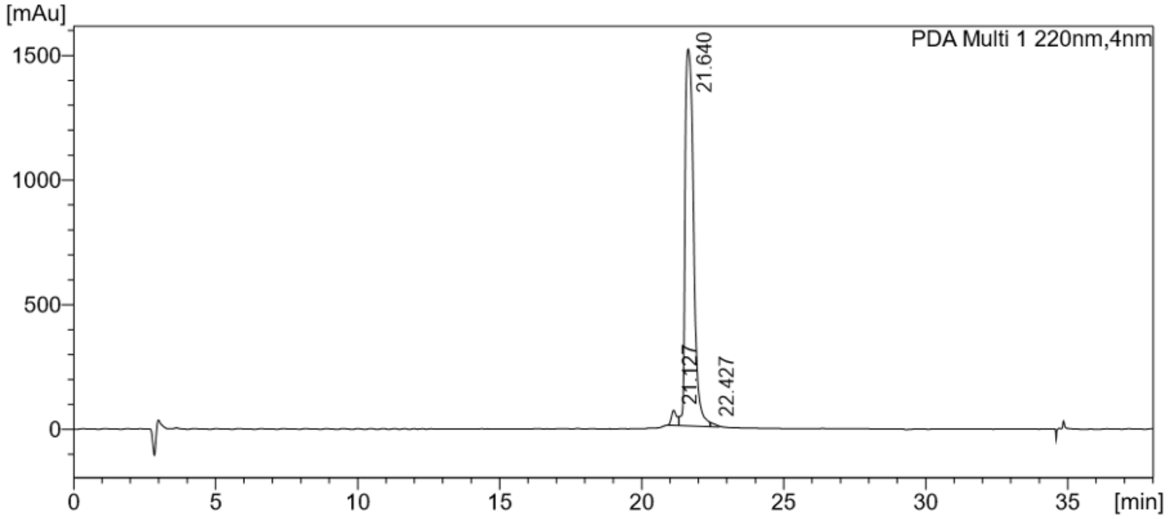

Figure S10. Analytical HPLC chromatogram of $(WRH)_4$.

**Peptide**	$(WRH)_4$
**Pump A**	0.1% Formic Acid in 100% Water
**Pump B**	0.1% Formic Acid in 100% Acetonitrile
**Total Flow**	0.4 ml/min
**Retention Time**	21.640 min
**% Purity**	96.495

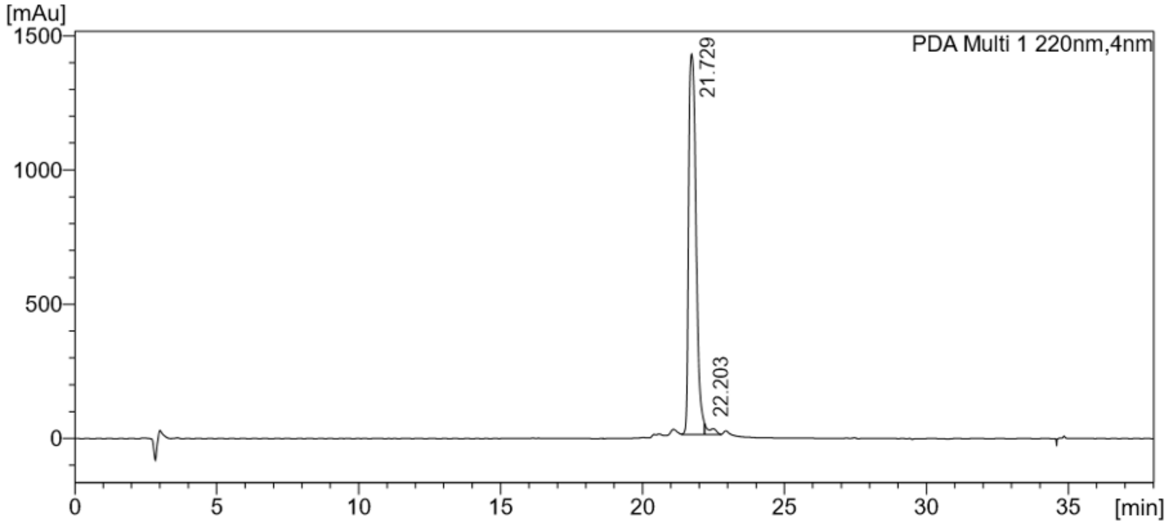

Figure S11. Analytical HPLC chromatogram of Oleyl-$(WRH)_3$.

**Peptide**	Oleyl-$(WRH)_3$
**Pump A**	0.1% Formic Acid in 100% Water
**Pump B**	0.1% Formic Acid in 100% Acetonitrile
**Total Flow**	0.4 ml/min
**Retention Time**	21.729 min
**% Purity**	97.318

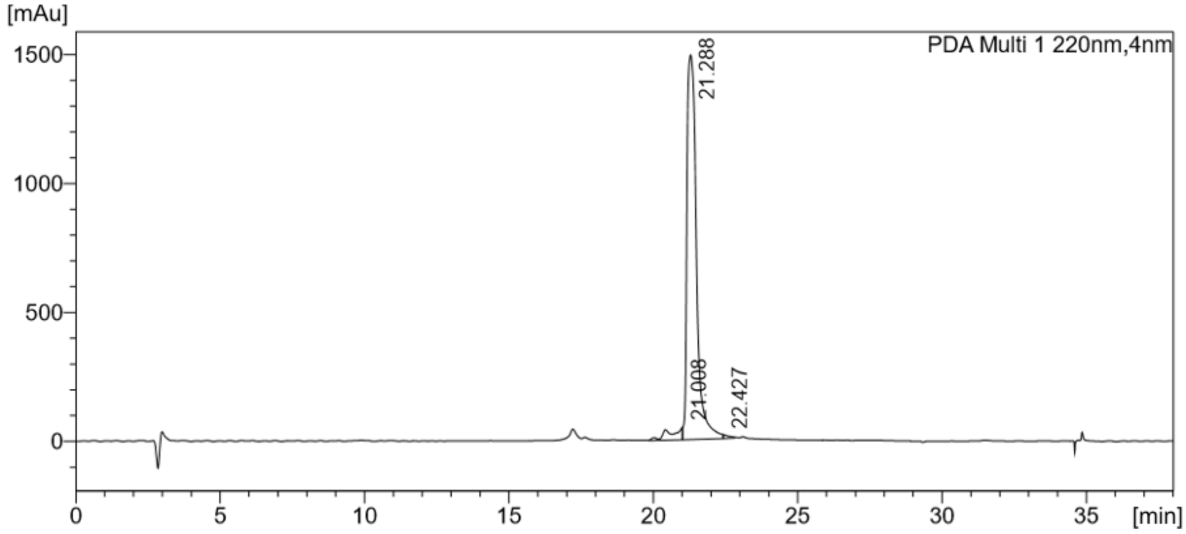

Figure S12. Analytical HPLC chromatogram of Oleyl-$(WRH)_4$.

**Peptide**	Oleyl-$(WRH)_4$
**Pump A**	0.1% Formic Acid in 100% Water
**Pump B**	0.1% Formic Acid in 100% Acetonitrile
**Total Flow**	0.4 ml/min
**Retention Time**	21.288 min
**% Purity**	95.359
